# Irisin Attenuates Pulmonary Vascular Remodeling in Pulmonary Arterial Hypertension via Ubiquitin‐Mediated Regulation of ENO1

**DOI:** 10.1002/advs.202500096

**Published:** 2025-06-25

**Authors:** Na Sun, Yong‐Bing Wang, Jing Huang, Run‐Wei Deng, Hui‐Yu He, Lei Gao, Xuan Gao, You‐Li Fan, Yan Cong, Yao‐Lei Guo, Yi‐Qiang Chen, Gui‐Jia Wang, Shao‐Hong Fang, Xia Gu, Bo Yu, Bing‐Xiang Wu

**Affiliations:** ^1^ Department of Cardiology Second Affiliated Hospital of Harbin Medical University Harbin 150086 China; ^2^ Key Laboratory of Myocardial Ischemia Ministry of Education Harbin Medical University Harbin 150086 China; ^3^ State Key Laboratory of Frigid Zone Cardiovascular Disease (SKLFCD) Harbin Medical University 246 Xuefu Road Harbin 150086 China; ^4^ Department of Cardiology First Affiliated Hospital of Harbin Medical University Harbin 150001 China; ^5^ Cardiovascular Imaging Center Second Affiliated Hospital of Harbin Medical University Harbin 150086 China

**Keywords:** irisin, enolase 1, pulmonary arterial hypertension, pulmonary artery smooth muscle cells, ubiquitination

## Abstract

Pulmonary vascular remodeling, which current therapeutic targets fail to alleviate disease severity, plays a key role in pulmonary arterial hypertension (PAH). Irisin is identified as a protective factor involved in regulating inflammation and oxidative stress, but its role in PAH remains unknown. To investigate, plasma irisin levels and its local pulmonary artery expression are measured in patients with PAH and mouse models. Irisin expression is significantly decreased in patients with PAH and PAH mouse models. Furthermore, overexpression and exogenous injection of irisin effectively alleviate hemodynamic and right‐heart function in PAH mouse models, meanwhile, it also reverses proliferation and cell cycle progression of pulmonary artery smooth muscle cells (PASMCs). To illustrate the mechanism of irisin exerts on PAH, Enolase 1 is identified as a key irisin‐interacting protein. Irisin suppresses proliferation of PDGF‐induced PASMCs by promoting ubiquitination status of Enolase 1 via E3 ligase of down regulated protein 4 in neural precursor cell development. Co‐immunoprecipitation and molecular docking analyses verifies the interaction and binding sites between irisin and its interactive proteins. Overall, these findings suggest that, irisin is a novel protective factor downregulated in PAH. By ubiquitination, irisin promotes Enolase 1 degradation and suppresses cell proliferation and pulmonary vascular remodeling in PAH.

## Graphical abstract

Irisin is a crucial plasma biomarker and promising therapeutic target that reflects disease severity, pulmonary vascular remodeling status and clinical outcome in patients with pulmonary arterial hypertension (PAH). As a novel protective factor, irisin is downregulated in PAH. By ubiquitination, irisin promotes Enolase 1 degradation and suppresses cell proliferation and pulmonary vascular remodeling in PAH.

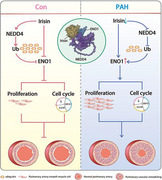

## 1 Introduction

Pulmonary arterial hypertension (PAH) is a progressive and life‐threatening disease in which the accumulation of hyper‐proliferative pulmonary artery smooth muscle cells (PASMCs) leads to obstruction of the pulmonary arteries, resulting in increased pulmonary artery pressure and right‐heart failure.^[^ ^1^ ^]^ Pulmonary vascular remodeling is the key pathogenesis in PAH. Current PAH therapies focus on vasodilation of partially occluded vessels, but fail to alleviate or reverse vascular remodeling lesions, which leaves poor prognosis of PAH.^[^ ^2^, ^3^, ^4^ ^]^ Therefore, it is critical to investigate effective therapeutic targets to improve pulmonary vascular remodeling conditions.

Irisin, a recently identified exercise‐induced myokine, contains 112 amino acids and is primarily secreted by skeletal and cardiac muscle cells.^[^ ^5^ ^]^ Small amounts of irisin are present in the brain, liver, adipose tissue, spleen, and stomach.^[^ ^6^ ^]^ Irisin is the product of type I membrane protein cleavage encoded by fibronectin type III domain‐containing 5 (FNDC5) genes.^[^ ^7^ ^]^ Since its discovery in 2012, several studies have shown that irisin participates in a variety of pathophysiological processes.^[^ ^8^ ^]^ Multiple studies have shown that plasma irisin levels are negatively correlated with the severity and prognosis of diseases such as COVID‐19, type 2 diabetes, and heart failure, with lower irisin levels indicating a higher risk of mortality.^[^ ^9^, ^10^, ^11^ ^]^ Furthermore, irisin demonstrates potential therapeutic value in cardiovascular diseases such as atherosclerosis, myocardial infarction, myocardial ischemia‐reperfusion injury, and heart failure.^[^ ^12^ ^]^ However, the role of irisin in PAH has rarely been investigated. Our previous study on irisin revealed an association between low plasma irisin levels and adverse clinical outcome in PAH, which has been a cohort validation for present study.^[^ ^13^ ^]^ However, a more intensive study on the effect of irisin on PAH clinical risk stratification and the detailed mechanism underlying the protective role of irisin deserves further investigation.

The glycolytic enzyme enolase 1 (ENO1), also known as 2‐phospho‐D‐glycerate hydrolase, is encoded by the ENO1 gene that localizes to chromosome 1p36.23.^[^ ^14^ ^]^ ENO1 plays a significant role in various cancers and autoimmune diseases, exerting effects of promoting proliferation and suppressing apoptosis.^[^ ^15^, ^16^, ^17^ ^]^ Hyper‐proliferation and resistance to apoptosis of PASMCs have been widely recognized to endow PAH a cancer‐like phenotype.^[^ ^18^ ^]^ However, whether the malignant phenotype associated with ENO1 is involved in the regulation of irisin levels in PAH remains unknown.

In the present study, we investigate the expression and functional roles of irisin in PAH. Our findings reveal that irisin expression is markedly reduced in the plasma and pulmonary arteries of patients with PAH. Overexpression of irisin in mouse PAH models suppresses pulmonary vascular remodeling, and alleviates hemodynamic parameters and right ventricular failure, indicating a protective role of irisin in PAH. In addition, irisin regulates the PAH phenotype mainly by down regulating of ENO1 via ubiquitination with the E3 ligase enzyme down regulated protein 4 in neural precursor cell development (NEDD4). Together, our findings give rise to the hypothesis for the first time that by interacting with ENO1, irisin may inhibit the proliferation and remodeling of PAH, which may in turn make irisin as a promising biomarker and a potential therapeutic target for PAH.

## 2 Results

### 2.1 Decreased Irisin Expression Correlates with Disease Severity and Poor Prognosis in Patients with PAH

**Figure 1:**
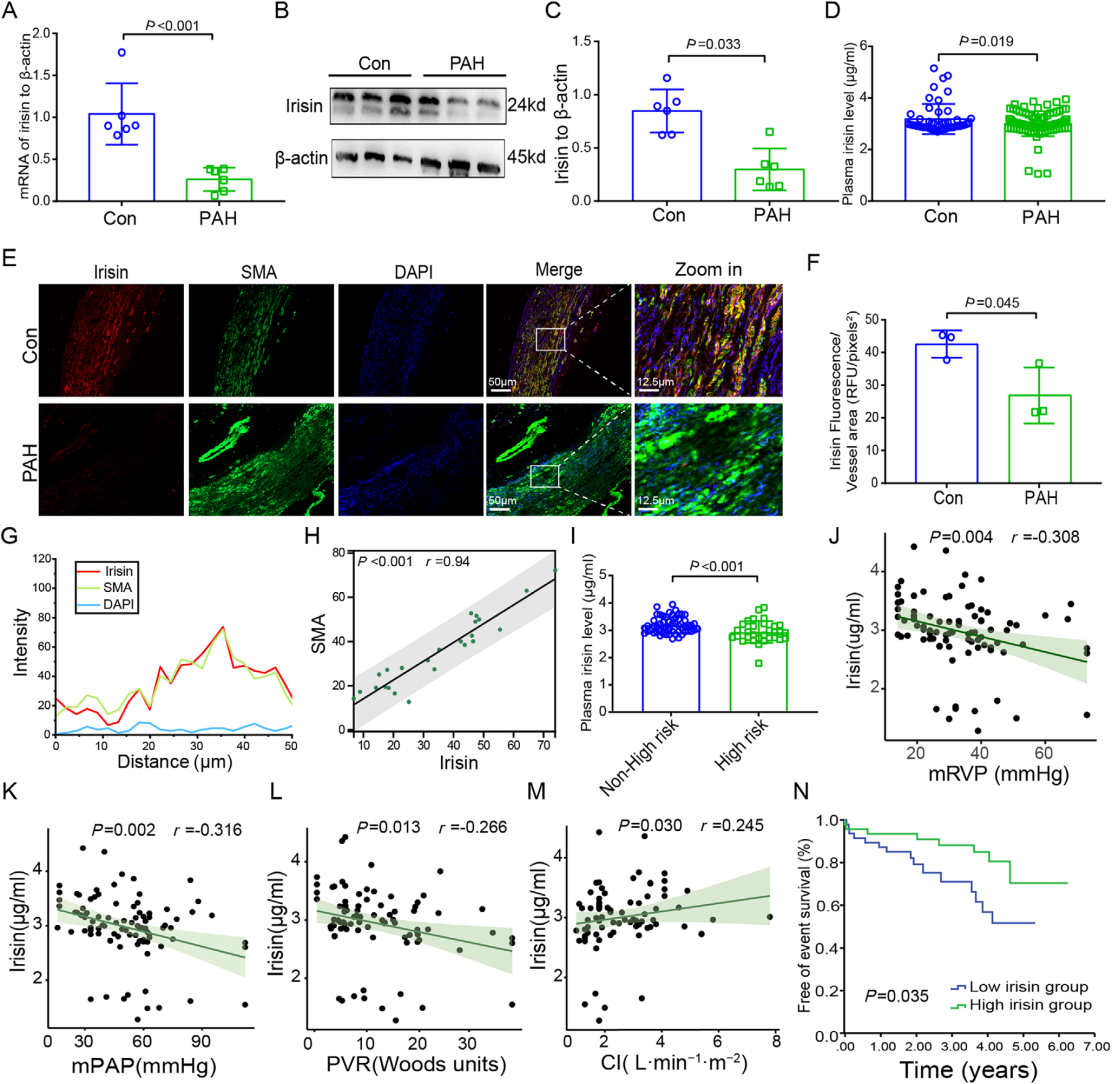
Reduced irisin expression in PAH patients and the clinical significance of irisin. A) Reduced mRNA expression of irisin in pulmonary arteries of PAH patients (*n* = 6). B,C) Decreased protein expression of irisin in pulmonary arteries of patients with PAH (*n* = 6). D) Reduced plasma irisin level in patients with PAH (*n* = 93) compared with control subjects (*n* = 50). E) Immunofluorescence analysis indicated the well‐co‐localization of irisin and SMA in pulmonary arteries of patients with PAH. F) Quantitative analysis illustrated the decreased expression level of irisin in pulmonary arteries of patients with PAH (*n* = 6). G) Co‐localization analysis between irisin and SMA in pulmonary arteries of control subjects. H) Pearson correlation analysis showed a positive correlation between irisin and SMA in pulmonary arteries of control subjects (*p* < 0.001, *R* = 0.94). I) Plasma irisin levels between non‐high‐risk (*n* = 55) and high‐risk(*n* = 38) patients with PAH. J) Correlation between plasma irisin level and mRVP (*p* = 0.004, *R* = −0.308). K) Correlation between plasma irisin level and mPAP (*p* = 0.002, *R* = −0.316). L) Correlation between plasma irisin level and PVR (*p* = 0.013, *R* = −0.266). M) Correlation between plasma irisin level and CI (*p* = 0.030, *R* = 0.245). N) Kaplan–Meier survival analysis indicates that low plasma irisin level predicts adverse clinical outcomes (*p* = 0.035). In all graphs, data are presented as mean ± SD. Data between 2 groups are compared by an independent‐sample two‐tailed Student's *t*‐test for (A)–(F), and (I).

We compare irisin expression in patients of Group 1 PAH with that in age‐ and sex‐matched controls. Reduced irisin mRNA (**Figure** **1**A) and protein (Figure 1B,C) expression are detected in the pulmonary arteries of patients with PAH. Immunofluorescence staining of pulmonary arteries from patients with PAH also shows low expression of irisin (Figure 1E,F). Using specific antibodies against smooth muscle actin (SMA), irisin is found to co‐localize with SMA with the Pearson's correlation analyze (*p* < 0.001, *R* = 0.94, Figure 1G,H). Bioinformatics analysis of the gene expression omnibus (GEO) datasets GSE144932 (*p* = 0.029) and GSE113439 (*p* = 0.005) also revealed a significant decrease in irisin (FNDC5) gene expression in patients with PAH (Figure S1, Supporting Information).

In addition, plasma irisin levels are significantly reduced in patients with PAH compared with that in the control group (Figure 1D), and patients with high‐risk stratification presented with even lower plasma irisin levels (Figure 1I), indicating the potential of irisin as a useful biomarker for PAH risk stratification. Patients with PAH are divided into two groups according to the median level (2.95 µg mL^−1^) of plasma irisin, and the baseline and hemodynamic parameters are compared in the two groups. Compared to the high‐irisin group, patients in the low‐irisin group have adverse cardiac function and hemodynamic parameters (Table S1, Supporting Information). As to the correlation analysis, we investigate the relationship among irisin and clinical, hemodynamic, patient's lifestyle and comorbidities. The plasma irisin levels are negatively correlated with mean right ventricular pressure (mRVP, *p* = 0.004, *R* = −0.308, Figure 1J), mean pulmonary artery pressure (mPAP, *p* = 0.002, *R* = −0.316, Figure 1K), pulmonary vascular resistance (PVR, *p* = 0.013, *R* = −0.266, Figure 1L), systolic PAP (*p* = 0.001, *R* = −0.330, Table S2, Supporting Information), dilated PAP (*p* = 0.007, *R* = −0.276, Table S2, Supporting Information), NT‐proBNP (*p* < 0.001, *R* = −0.503, Table S2, Supporting Information) and WHO cardiac function (*p* = 0.023, *R* = −0.236, Table S2, Supporting Information). In addition, irisin is positively correlated with cardiac index (CI, *p* = 0.030, *R* = 0.245, Figure 1M) and 6 min walking distance (6MWD, *p* = 0.012, *R* = 0.258, Table S2, Supporting Information). Other correlated parameters were presented in Table S2 (Supporting Information). All recruited patients undergo regular follow‐up. During the follow‐up period, 23 patients (24.7%) experienced clinical worsening events, including 8 patients (17.4%) in the high‐irisin group and 15 (31.9%) in the low‐irisin group. Kaplan–Meier survival analysis demonstrates that patients with PAH and lower plasma irisin levels have poor prognosis (*p* = 0.035, Figure 1N). In univariate and multivariate Cox proportional regression analyses, plasma irisin level is found to be an independent predictor of clinical worsening events (hazards ratios [HRs], 0.479; confidence intervals [CIs], (0.277, 0.830), *p* = 0.009, Table S3, Supporting Information).

### 2.2 Irisin Expression Is Reduced in the Pulmonary Vasculature in Experimental Models of PAH

**Figure 2:**
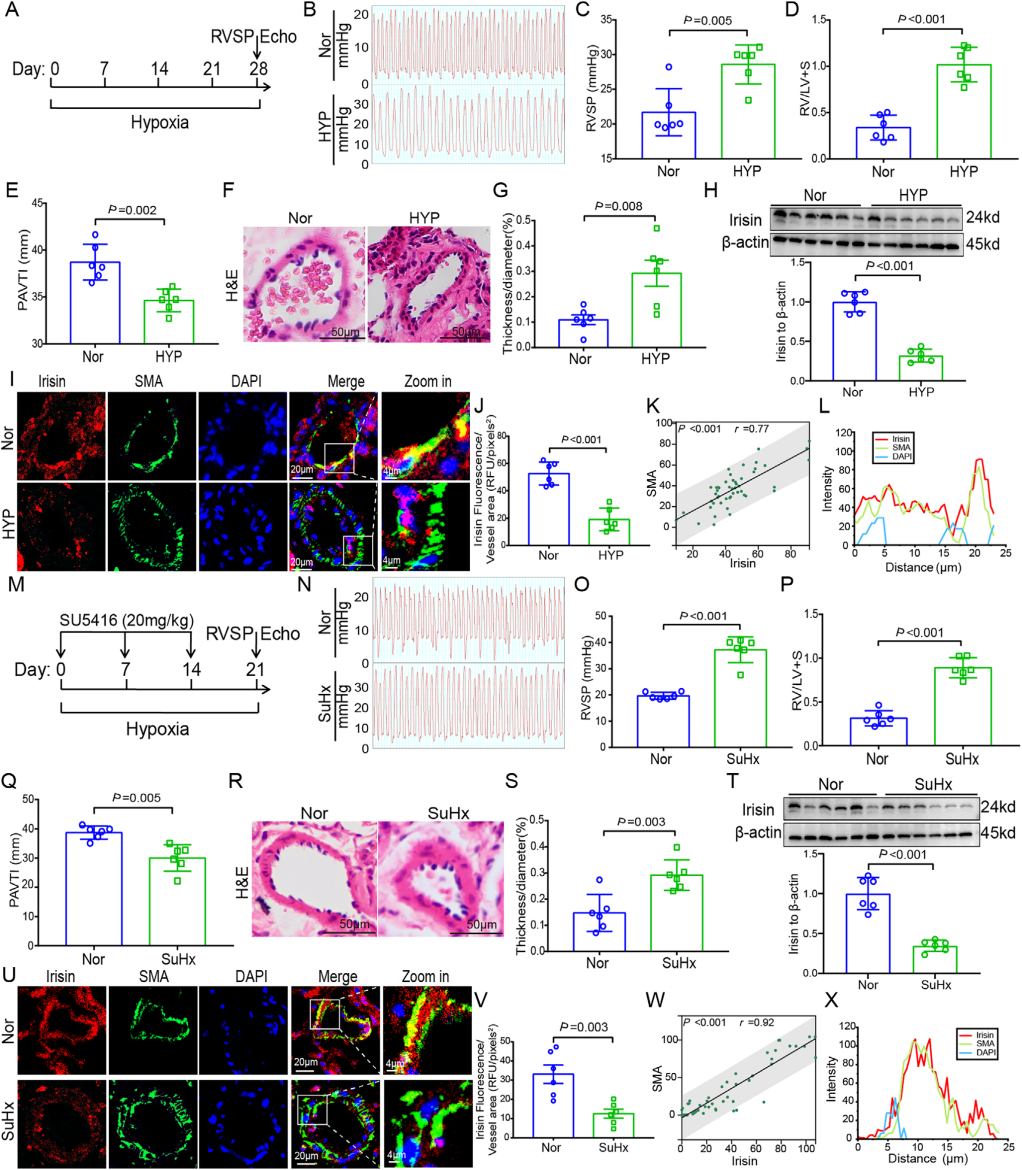
Decreased irisin expression level in PAH mouse models of hypoxia and SuHx. A) Schematic representation of hypoxia mouse model. B) Representative images of RVSP in hypoxia and control mice. C) Quantitative analysis RVSP of hypoxia and control mice (*n* = 6). D) Increased RV/LV+S ratio of hypoxia mice (*n* = 6). E) Decreased right ventricular function of PAVTI in hypoxia mice (*n* = 6). F) Images of H&E staining of hypoxia mice revealed the exacerbated pulmonary vascular remodeling. G) Quantitative analysis indicates the increased thickness of pulmonary vasculature in hypoxia mice (*n* = 6). H) Western Blot reveals the reduced expression of irisin in the lungs of hypoxia mice (*n* = 6). I) Immunofluorescence images of irisin (Red) and SMA (Green) expression in lungs of hypoxia mice and normoxia mice. J) Quantitative analysis illustrates the decreased expression level of irisin in the lungs of hypoxia mouse model (*n* = 6). K) Pearson correlation analysis shows a positive correlation between irisin and SMA expression in pulmonary arteries. L) Immunofluorescence analysis indicates the well co‐localization of irisin and SMA in lungs of a normoxia mouse model. M) Schematic representation of SuHx mouse model. N) Representative images of RVSP in SuHx and control mice. O) Quantitative analysis RVSP of SuHx and control mice (*n* = 6). P) Increased RV/LV+S ratio of SuHx mice (*n* = 6). Q) Decreased right ventricular function of PAVTI in SuHx mice (*n* = 6). (R) Images of H&E staining of SuHx mice reveal the exacerbated pulmonary vascular remodeling. S) Quantitative analysis indicates the increased thickness of pulmonary vasculature in SuHx mice (*n* = 6). T) Western Blot reveals the reduced expression of irisin in lungs of SuHx mice (*n* = 6). U) Immunofluorescence images of irisin (Red) and SMA (Green) expression in lungs of SuHx mice and normoxia mice. V) Quantitative analysis illustrates the decreased expression level of irisin in the lungs of SuHx mice model (*n* = 6). W) Pearson correlation analysis shows a positive correlation between irisin and SMA expression in pulmonary arteries. X) Immunofluorescence analysis indicates the well co‐localization of irisin and SMA in the lungs of normoxia mouse model. In all graphs, data are presented as mean ± SD. Data between 2 groups are compared by an independent‐sample two‐tailed Student's *t*‐test for (B)–(J) and (N)–(V).

We examine the expression pattern of irisin in two experimental models of PAH: a hypoxia and a SU5416/hypoxia (SuHx, 20 mg kg^−1^) mouse model. The success of the hypoxia mouse model is examined by hemodynamic and other related parameters (**Figure** **2**A–G; Table S4, Supporting Information). Compared with normoxia mouse, hypoxia mouse model shows higher right ventricular systolic pressure (22.03±3.41 mmHg v.s. 28.30±2.72 mmHg, *p* = 0.005, RVSP), elevated ratio of right ventricular to left ventricular + septum (0.34±0.14 v.s. 1.02±0.19, *p* < 0.001, RV/LV+S), lower pulmonary artery velocity time integral (38.71±1.91 mm v.s. 34.51±1.44 mm, *p* = 0.002, PAVTI) and increased pulmonary vasculature (0.11±0.05 mm v.s. 0.29±0.13 mm, *p* = 0.008). Hypoxia mouse exhibits reduced irisin expression in the lungs compared with normoxia controls (Figure 2H–J). Compared to normoxia mouse, irisin expression level in the lung of hypoxia mouse is reduced by nearly 68.0% evaluated by Western Blot and decreased by nearly 63.6% assessed by immunofluorescence. Immunofluorescence analysis reveals good co‐localization of irisin and SMA marker with the Pearson's correlation analysis in hypoxia mouse model (*p* < 0.001, *R* = 0.77, Figure 2K,L). Similarly, SuHx mouse model also exhibits reduced irisin expression (Figure 2M–V) and well co‐localization of irisin with SMA in the lungs (*p* < 0.001, *R* = 0.92, Figure 2W,X). In the lung tissue of SuHx mouse, irisin expression level reduces nearly 65.1% assessed by Western Blot and 49.1% evaluated by immunofluorescence. By comparing with normoxia controls, irisin expression decreased in the lungs of both hypoxia and SuHx mice (Figure S2A,B, Supporting Information). Detailed hemodynamic data between hypoxia mouse model and SuHx mouse model are shown in Table S4 (Supporting Information).

### 2.3 Irisin Overexpression in Mice Reverses Hypoxia and SuHx‐Induced PAH

**Figure 3:**
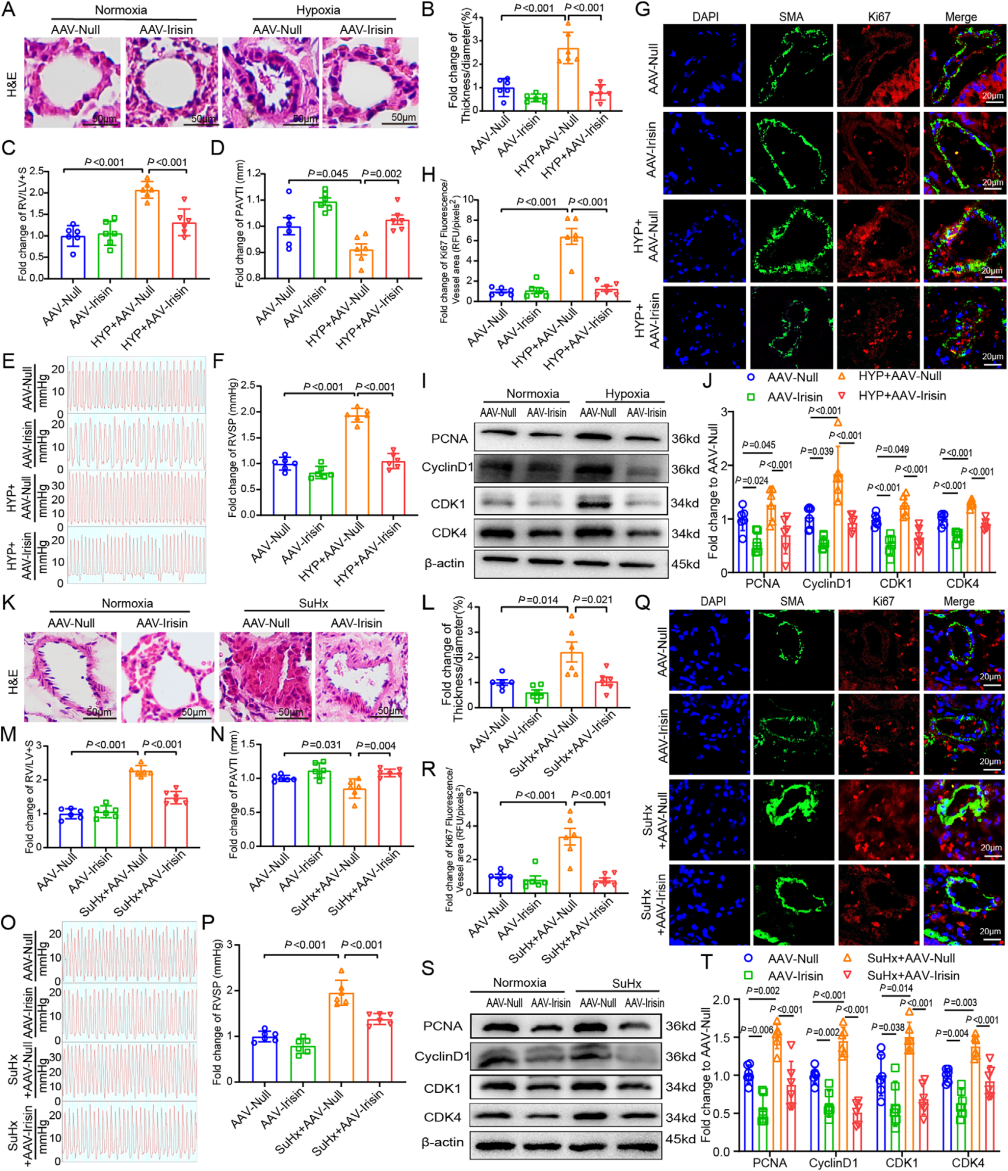
Irisin overexpression in mice reverses hypoxia and SuHx induced PAH. A) Images of H&E staining of AAV‐irisin overexpression reveals the improved pulmonary vascular remodeling in hypoxia mice. B) Quantitative analysis indicates the reduced thickness of pulmonary vasculature in AAV‐irisin overexpression mice (*n* = 6). C) Decreased RV/LV+S ratio of AAV‐irisin overexpression mice (*n* = 6). D) Increased right ventricular function of PAVTI in AAV‐irisin overexpression mice (*n* = 6). E) Representative images of right heart catheterization of hypoxia and normoxia mice with AAV‐irisin overexpression or not. F) Quantitative analysis indicates reduced RVSP in AAV‐irisin overexpression mice (*n* = 6). G) Immunofluorescence images of Ki67 (Red) and SMA (Green) expression in the lungs of different mice groups. H) Quantitative analysis of immunofluorescence illustrates the decreased Ki67 expression in AAV‐irisin overexpression mice (*n* = 6). I,J) Western Blot demonstrates the decreased protein expression of PCNA, CyclinD1, CDK1, and CDK4 (*n* = 6). K) Images of H&E staining of AAV‐irisin overexpression revealed the improved pulmonary vascular remodeling in SuHx mice. L) Quantitative analysis indicates the reduced thickness of pulmonary vasculature in AAV‐irisin overexpression mice (*n* = 6). M) Decreased RV/LV+S ratio of AAV‐irisin overexpression mice (*n* = 6). N) Increased right ventricular function of PAVTI in AAV‐irisin overexpression mice (*n* = 6). O) Representative images of right heart catheterization of SuHx and control mice with AAV‐irisin overexpression or not. P) Quantitative analysis indicates reduced RVSP in AAV‐irisin overexpression mice (*n* = 6). Q) Immunofluorescence images of Ki67 (Red) and SMA (Green) expression in the lungs of different mice groups. R) Quantitative analysis of immunofluorescence illustrates the decreased Ki67 expression in AAV‐irisin overexpression mice (*n* = 6). S,T) Western Blot demonstrates the decreased protein expression of PCNA, CyclinD1, CDK1 and CDK4 (*n* = 6). In all graphs, data are presented as mean ± SD. Data among 4 groups are compared by a two‐way ANOVA test followed by a Tukey post hoc test for (A)–(T).

To determine the in vivo effect of irisin on PAH pathogenesis and hemodynamic and right ventricular functions, an adeno‐associated virus 5 (AAV5)‐irisin overexpression vector is used to infect a PAH mouse model. In the hypoxia mouse model, the AAV‐irisin overexpression mice show decreased 70.3% wall thickness of pulmonary arteries, indicating alleviated pulmonary vascular remodeling, compared with hypoxia mice (**Figure** **3**A,B). The ratio of RV/LV+S is reduced by 36.5% in the irisin overexpressing mice compared with that in the hypoxia mice (Figure 3C), which suggests improved right ventricular function in the irisin overexpressing mice. Echocardiography demonstrates increased PAVTI of 11.1% in irisin overexpressing mice compared with that in the hypoxia mouse model (Figure 3D). AAV‐irisin overexpressing mice reduce 45.8% RVSP compared with hypoxia mouse model (Figure 3E,F). To better investigate the effect of irisin overexpression on cardiac function, we evaluated the tricuspid annular plane systolic excursion (TAPSE), which indicates an increased TAPSE value in irisin‐overexpressing mice compared with that in the hypoxia mouse model (Figure S2C, Supporting Information).

Overexpression of irisin also suppresses cell proliferation and cell cycle progression in a PAH mouse model. Proliferation markers, including proliferating cell nuclear antigen (PCNA) and Ki67, were significantly decreased in irisin overexpression mice compared with those in the hypoxia mice (Figure 3G–J). In addition, cell cycle‐associated proteins, including cyclin D1, cyclin‐dependent kinase 1 (CDK1) and cyclin‐dependent kinase4 (CDK4), are also markedly reduced (Figure 3I,J) in the irisin overexpressing mice, demonstrating impairment of cell cycle progression, which further results in the inhibition of proliferation. We also verified the above results in the SuHx PAH mouse model and obtained a similar conclusion to that obtained from the hypoxia mouse model (Figure 3K–T). The detailed numerical data are presented in Table S5 (Supporting Information).

In addition, we investigated the fibrotic changes by evaluating Collagen I expression level and Masson's trichrome staining. Overexpression of irisin reduces Collagen I expression in both hypoxia and SuHx mouse models (Figure S2D–G, Supporting Information). Masson's trichrome staining reveals significant collagen deposition in the pulmonary vascular wall in hypoxia and SuHx mouse, while irisin overexpression may alleviate collagen deposition (Figure S2H–K, Supporting Information).

Furthermore, we evaluate the effect of irisin on metabolic changes as well. Hypoxia mice exhibit lower body weight and reduced blood glucose, while overexpression of irisin may reverse the above alterations (Figure S2L,M, Supporting Information).

### 2.4 Irisin Suppresses Proliferation and Cell Cycle Progression in PASMCs

The results of the in vivo experiments described above suggest that irisin may inhibit proliferation and pulmonary vascular remodeling in PAH mice. Therefore, we hypothesize that irisin would mediate similar functions in vitro. Immunofluorescence staining reveals that irisin is distributed in the cytoplasm, nucleus, and cell membrane, which shows good co‐localization with SMA (Figure S3A,B, Supporting Information). Platelet‐derived growth factor (PDGF), an effective inducer of cell proliferation, is widely used in in vitro studies of PAH. We examined the mRNA expression of PDGF genes in the pulmonary arteries of patients with PAH and found elevated mRNA expression of PDGF in the patients (Figure S3C, Supporting Information); therefore, we applied PDGF‐treated PASMCs as the in vitro cell model. PASMCs are treated with PDGF (50 ng mL^−1^) for 24 and 48 h. Irisin expression is found to be significantly reduced in the 48 h treatment group (Figure S3D,E, Supporting Information). In addition, we investigate the effect of different cell‐level gradients on irisin expression. The results show that within a certain range of cell levels, the expression of irisin decreased with increasing cell levels; however, when the cell levels reach a large amount, the trend of change is no longer significant (Figure S5G,H, Supporting Information). The above phenomenon indicates potential feedback mechanism may play a role in the process, which needs further investigation.

**Figure 4:**
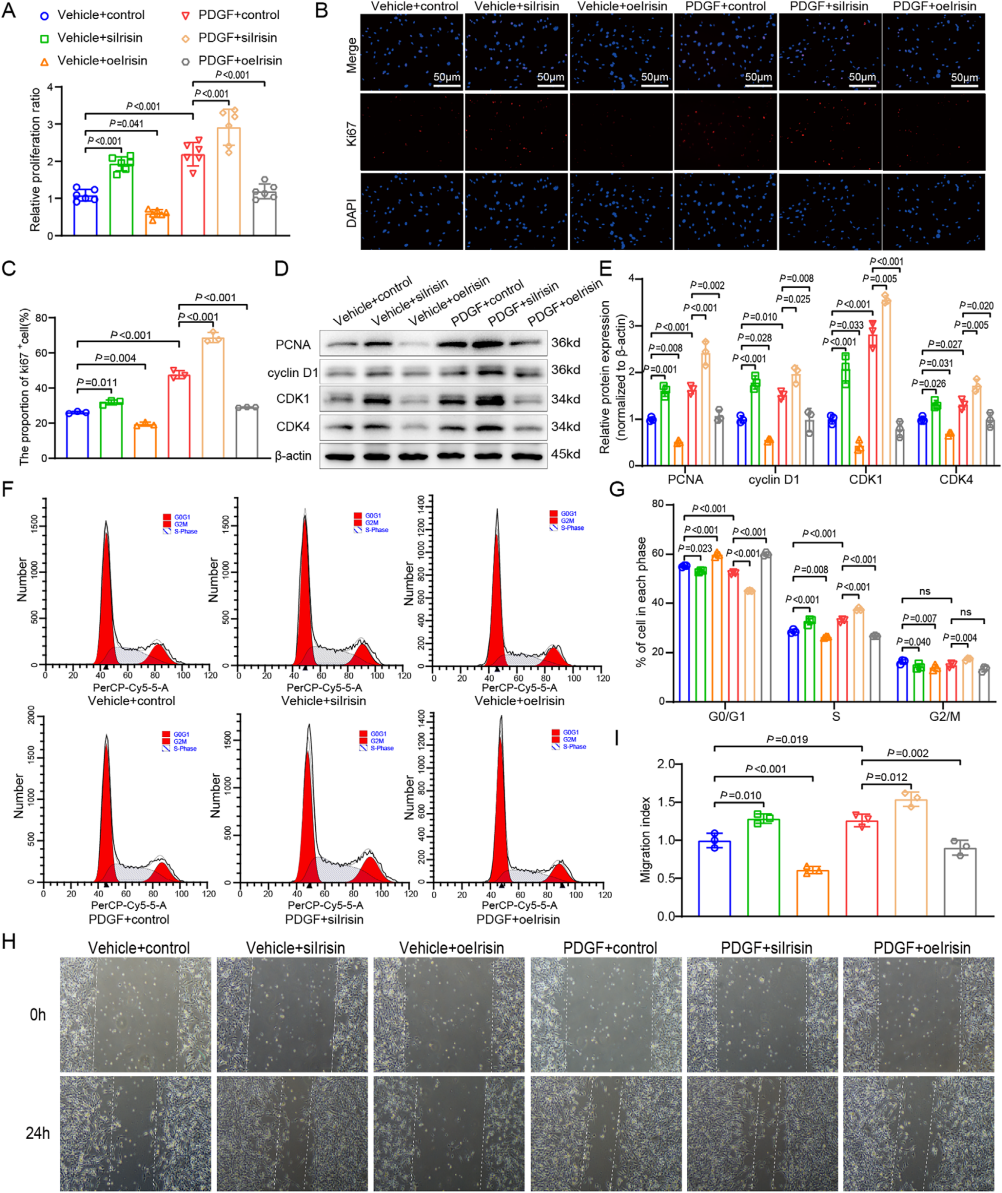
Irisin suppresses the proliferation and cell cycle progression of PDGF‐induced PASMCs. A) CCK8 shows that irisin overexpression inhibits PDGF‐induced PASMCs proliferation, whereas irisin knockdown exerts the opposite effect. (*n* = 6). B,C) Overexpression of irisin inhibits the expression of Ki67, whereas irisin knockdown indicates the opposite effect. (*n* = 3). D,E) Overexpression of irisin suppresses the expression of PCNA, CyclinD1, CDK1 and CDK4, whereas irisin knockdown shows the opposite effect. (*n* = 3). F) Flow cytometry cell cycle analysis of different irisin overexpression cell groups in PASMCs. G) Irisin overexpression decreases G0/G1 phase and G2/M phase proportion in PDGF‐induced PASMCs, whereas irisin knockdown has the opposite effect. (*n* = 3). H,I) Scratch experiments show that irisin overexpression inhibits PDGF‐induced PASMCs migration, whereas irisin knockdown has the opposite effect. (*n* = 3). In all graphs, data are presented as mean ± SD. Data among 6 groups were compared by a two‐way ANOVA test followed by a Tukey post hoc test for (A)–(I).

Enrichment analysis of the GSE113439 dataset reveals a negative correlation between irisin and cell cycle regulation (Figure S3F, Supporting Information). Therefore, we further investigate the effect of irisin on PASMCs' proliferation and cell cycle. Results of the Cell Counting Kit 8 (CCK8) assay demonstrate that overexpression of irisin effectively inhibits PDGF‐induced cell proliferation, conversely, irisin knockdown promotes cell proliferation (**Figure** **4**A). Furthermore, irisin overexpression decreases PCNA (*p* = 0.002) and Ki67 expression (*p* < 0.001), whereas irisin knockdown increases the expression of proliferation proteins of PCNA (*p* < 0.001) and Ki67 (*p* < 0.001, Figure 4B–E), indicating the proliferation suppressive ability of irisin. We also verify the proliferation ability of irisin by EdU experiment and obtain similar results (Figure S4A,B, Supporting information). Western blot analysis of cell cycle markers reveals that irisin overexpression reduces cyclinD1, CDK1 and CDK4 expression, and knockdown of irisin increases the expression of these cell cycle proteins (Figure 4D,E). Flow cytometric analysis demonstrates that fewer PASMCs remain in the G0/G1 and G2/M phases in irisin overexpressing cells; conversely, irisin knockdown increases the number of G0/G1 and G2/M phase cells (Figure 4F,G), which is consistent with the cell cycle protein expression pattern. We also verify the effect of irisin on mouse PAMSCs and obtain similar results. In mouse PASMCs, the expression of irisin is markedly reduced in PDGF‐treated cells for 48 h (Figure S5A,B, Supporting Information). In addition, exogenous irisin treatment suppresses the proliferation of human and mouse PASMCs in a concentration‐dependent manner (Figure S5C–F, Supporting Information).

In addition, the effects of irisin on other factors are evaluated, including migration, contraction, synthesis and resistance to apoptosis in PASMCs. First, overexpression of irisin effectively inhibits PDGF‐induced cell migration, conversely, irisin knockdown promotes cell migration (Figure 4H,I). Second, in the examination of apoptosis markers, overexpression of irisin increases Bax expression, while reducing Bcl‐2 and Caspase‐3 expression, and knockdown of irisin may exert the opposite effect (Figure S4C,D, Supporting Information). Furthermore, as to the contraction and synthesis markers, overexpression of irisin may decrease α‐SMA expression, meanwhile elevate OPN expression; conversely, knockdown of irisin reverses the results (Figure S4E,F, Supporting Information).

### 2.5 Irisin Interacts with ENO1 and Recruits E3 Ubiquitin Ligase NEDD4 to Regulate ENO1 Protein Stability

**Figure 5:**
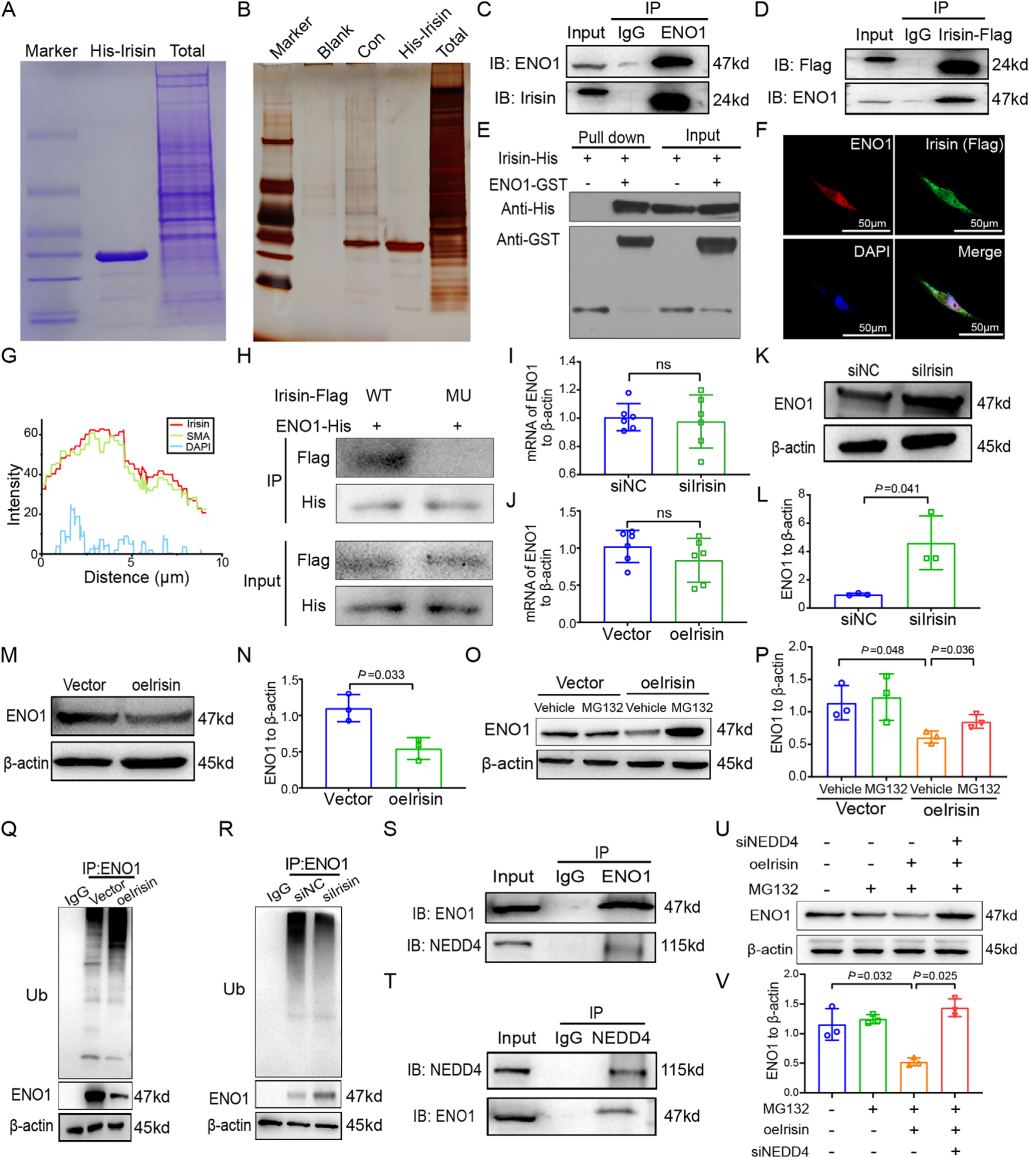
Irisin interacts with ENO1 and recruits E3 ubiquitin ligase NEDD4 to regulate ENO1 protein stability. A,B) Pull‐down staining images of irisin and ENO1 are identified by mass spectrometric analysis. C,D) Interaction between irisin and ENO1 is confirmed by immunoprecipitation and Western Blot. E) Pull‐down assay verifies the direct interaction between irisin and ENO1. F) Immunofluorescence images present the interaction between irisin‐Flag (Green) and ENO1 (Red). G) Immunofluorescence analysis indicates the well co‐localization of irisin and ENO1 in PASMCs. H) Co‐IP experiments confirm the loss of the interaction between irisin and ENO1 when the core amino acid bound to ENO1 is mutated to alanine. I,J) RT‐qPCR results show that irisin knockdown or overexpression has no effect on ENO1 mRNA expression. K–N) Western blot results show that irisin knockdown promotes ENO1 expression, while irisin overexpression has the opposite effect. O,P) MG132 treatment increases ENO1 expression in irisin overexpressing PASMCs (*n* = 3). Q) Overexpression of irisin enhances ubiquitination of ENO1. R) Knockdown of irisin decreases ubiquitination of ENO1. S,T) Interaction between ENO1 and NEDD4 is confirmed by immunoprecipitation and Western Blot. (U and V) Knockdown of NEDD4 further increases the ENO1 expression in PASMCs treated with MG132 and irisin overexpression (*n* = 3). In all graphs, data are presented as mean ± SD. Data between 2 groups are compared by an independent‐sample two‐tailed Student's *t*‐test for (I)–(N). Data among 4 groups are compared by a two‐way ANOVA test followed by a Tukey post hoc test for (O)–(P), and (U)–(V).

Co‐immunoprecipitation (Co‐IP) and pull‐down assays are performed to investigate the mechanisms by which irisin regulates the proliferation and cell cycle in PASMCs. Purified His‐irisin fusion protein is incubated with PASMCs, and mass spectrometry is performed (**Figure** **5**A,B). ENO1 is identified as an irisin‐interacting protein. A Co‐IP assay is performed to confirm the interaction between irisin and ENO1 (Figure 5C,D). Pull‐down assays with purified fusion proteins His‐irisin and GST‐ENO1 were performed to further confirm their direct interaction in vitro (Figure 5E). Immunofluorescence also reveals the co‐localization of irisin and ENO1 in PASMCs (Figure 5F,G).

To understand the structural basis of the interaction between irisin and ENO1, we perform protein‐protein docking analysis. We select the configuration with the most stable protein binding among 70000 calculations, the energy of which is ←500 kcal mol^−1^. ASN407 of ENO1 and GLN108 of irisin appear to form a hydrogen bond, whereas ARG399 of ENO1 and GLN78 of irisin form two hydrogen bonds. In addition, TYR188 of ENO1 and ASN81 of irisin also form a hydrogen bond, which forms the basic structure of the interaction between irisin and ENO1 (Figure S6A, Supporting Information). According to the analysis, we identify the core amino acids of irisin that interact with ENO1, including LEU74, GLN78, ASN81, GLN103, and GLN108. In order to verify the amino acids of irisin that interact with ENO1, we mutated the above five amino acids into alanine and further examined the interaction ability between irisin and ENO1. The results indicate that irisin and ENO1 are unable to interact with each other after mutation of the above amino acids (Figure 5H). Detailed information on the binding sites is presented in Table S6 (Supporting Information).

As irisin interacts with ENO1, we sought to determine whether irisin is involved in the regulation of ENO1 expression. Western blotting reveals that ENO1 protein expression is significantly upregulated following irisin knockdown (Figure 5K,L), whereas ENO1 mRNA expression remained unaffected (Figure 5I). Conversely, ENO1 protein expression is down‐regulated following irisin overexpression, with ENO1 mRNA expression unchanged (Figure 5J,M,N). To determine whether irisin regulates ENO1 expression at the post‐transcriptional level, cells are treated with MG132 (an inhibitor of the ubiquitin proteasome pathway). Treatment of irisin overexpressing cells with MG132 leads to marked accumulation of ENO1 protein, indicating that irisin regulates ENO1 protein stability (Figure 5O,P). As ubiquitination is a common mechanism of protein degradation, we further investigate whether irisin induces ENO1 degradation by promoting ubiquitination in PASMCs. ENO1 is immunoprecipitated with anti‐ENO1 antibodies, and its ubiquitination level is determined using an anti‐ubiquitin antibody. Irisin overexpression markedly increases the ENO1 ubiquitination (Figure 5Q), conversely, knockdown of irisin decreased ENO1 ubiquitination (Figure 5R).

To investigate the mechanism of irisin‐mediated degradation of ENO1 protein, we use UbiBrowser (http://ubibrowser.bio‐it.cn/ubibrowser/) to predict the E3 ubiquitin ligase that may interact with both ENO1 and irisin, we examine the likely proteins ranking top three (NEDD4, CBL, and SYVN1) and use Co‐IP method, which identifies NEDD4 as the most promising candidate (Figure S6C,D, Supporting Information). Co‐IP is performed to determine whether irisin‐dependent degradation of ENO1 is mediated by NEDD4. As expected, NEDD4 is found to interact with ENO1 (Figure 5S,T). In addition, knockdown of NEDD4 further enhanced ENO1 expression in MG132‐treated PASMCs that overexpress irisin (Figure 5U,V), which confirms that irisin serves as a scaffold to recruit NEDD4 to regulate ENO1 protein stability.

A molecular docking study is performed to further determine the binding conformation of irisin, ENO1, and NEDD4. The results demonstrate that irisin, ENO1, and NEDD4 bind with one another to form a stable structure (Figure S6B, Supporting Information). GLU57 of irisin and ENO1 form a salt bridge with LYS951 of NEDD4, whereas LYS27 of irisin and ENO1 form a hydrogen bond with THR902 of NEDD4. Furthermore, ARG178 of irisin and ENO1 form 27 van der Waals bonds with HIS878 of NEDD4, which constitutes the structural foundation of the stable conformation of irisin, ENO1, and NEDD4 (Table S7, Supporting Information).

### 2.6 Irisin Alleviates Proliferation and Cell Cycle in PASMCs by Targeting ENO1

**Figure 6:**
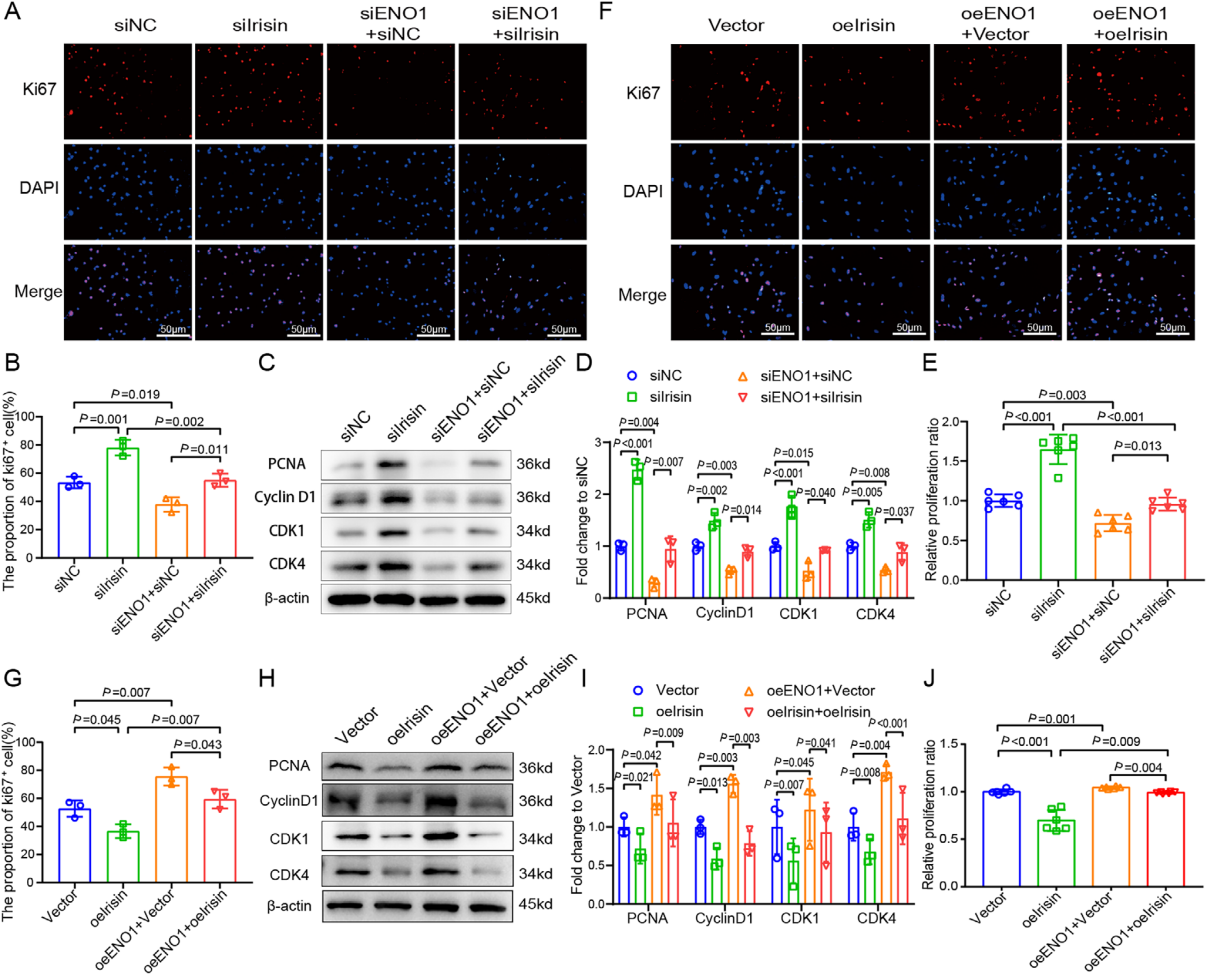
Irisin alleviates proliferation and cell cycle in PASMCs by targeting ENO1. A,B) Immunofluorescence assays reveal that the enhancement of Ki67 expression following irisin knockdown is reversed by ENO1 knockdown (*n* = 3). C,D) The increased protein expression of PCNA, CDK1, CDK4, and CyclinD1 following the knockdown of irisin is reversed by ENO1 knockdown (*n* = 3). E) The CCK8 assay demonstrates that the enhancement of cell proliferation induced by irisin knockdown is reversed by ENO1 knockdown (*n* = 6). F,G) Immunofluorescence assays reveal that the reduction of Ki67 expression following irisin overexpression is reversed by ENO1 overexpression (*n* = 3). H,I) The decreased protein expression of PCNA, CDK1, CDK4, and CyclinD1 following the overexpression of irisin is reversed by ENO1 overexpression (*n* = 3). J) The CCK8 assay demonstrates that the reduction of cell proliferation induced by irisin overexpression is reversed by ENO1 overexpression (*n* = 6). In all graphs, data are presented as mean ± SD. Data among 4 groups are compared by two‐way ANOVA test followed by Tukey post hoc test for (A)–(J).

To investigate whether ENO1 is involved in the irisin‐mediated regulation of proliferation and cell cycle, we alter the expression levels of irisin and ENO1 in PASMCs. Irisin knockdown promotes cell proliferation and increases proliferation indicator Ki67 and PCNA expression, which can be rescued by ENO1 knockdown (**Figure** **6**A–E; Figure S7A,B, Supporting Information). Whereas, overexpression of irisin inhibits cell proliferation and reduces expression of Ki67 and PCNA, which can be reversed by ENO1 overexpression (Figure 6F–J; Figure S7C,D, Supporting Information). In addition, irisin knockdown in siENO1‐treated cells increases the expression of cell cycle proteins, including cyclinD1, CDK1 and CDK4 (Figure 6C,D). Accordingly, overexpression of irisin in ENO1 overexpressing cells decreases the expression of PCNA and cell cycle proteins (Figure 6H,I). These results indicate that irisin suppresses the proliferation and cell cycle of PASMCs via ENO1.

To better understand the relationship between irisin and ENO1‐mediated proliferation arrest, we reintroduce ENO1 after its siRNA reduction in irisin‐lacking cells. In irisin‐deficient cells, knockdown of ENO1 attenuates the proliferative effect of irisin on PASMCs, which is subsequently restored by re‐expression of ENO1. The proliferation evaluation is conducted through immunofluorescence of Ki67, EdU, CCK8, and Western Blot examination of proliferation and cell cycle markers (Figure S8, Supporting Information).

### 2.7 Irisin Supplementation Prevents Hypoxia and SuHx PAH in Mice

**Figure 7:**
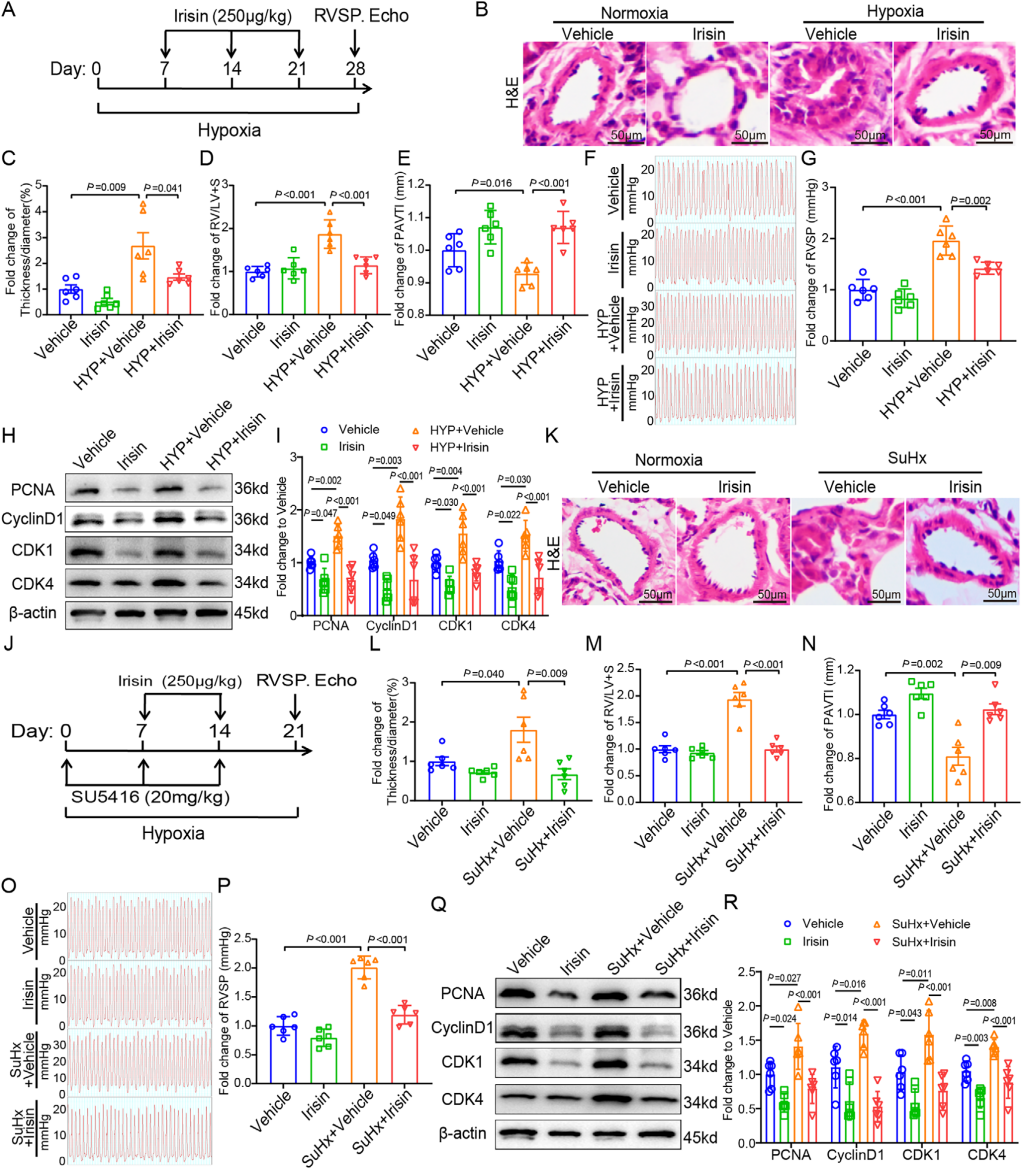
Irisin supplement to mice prevents hypoxia and SuHx‐induced PAH. A) Schematic representation of irisin injection to hypoxia mouse model. B) Images of H&E staining of exogenous irisin injection (250 µg kg^−1^ per week) reveal the improved pulmonary vascular remodeling in hypoxia mice. C) Quantitative analysis indicate the reduced thickness of pulmonary vasculature in irisin injection mice (*n* = 6). D) Decreased RV/LV+S ratio of irisin injection mice (*n* = 6). E) Increased PAVTI in irisin injection mice (*n* = 6). F) Representative images of RVSP in different mice groups. G) Quantitative analysis indicates reduced RVSP in irisin injection mice (*n* = 6). H,I) Western Blot demonstrates the decreased protein expression of PCNA, CyclinD1 and CDK1, CDK4 (*n* = 6). J) Schematic representation of irisin injection to SuHx mouse model. K) Images of H&E staining of exogenous irisin injection (250 µg kg^−1^ per week) reveal the improved pulmonary vascular remodeling in SuHx mice. L) Quantitative analysis indicates the reduced thickness of pulmonary vasculature in irisin injection mice (*n* = 6). M) Decreased RV/LV+S ratio of irisin injection mice (*n* = 6). N) Increased PAVTI in irisin injection mice (*n* = 6). O) Representative images of RVSP in different mice groups. P) Quantitative analysis indicates reduced RVSP in irisin injection mice (*n* = 6). Q,R) Western Blot demonstrates the reduced protein expression of PCNA, CyclinD1 and CDK1, CDK4 (*n* = 6). In all graphs, data are presented as mean ± SD. Data among 4 groups are compared by a two‐way ANOVA test followed by a Tukey post hoc test for (B)–(I) and (K)–(R).

As irisin effectively suppresses the proliferation and cell cycle progression of PASMCs, we further investigate the beneficial effects of supplementing PAH mice with exogenous irisin. Upon the pre‐experiment of irisin dose gradient (Figure S9, Supporting Information) and literature investigation, 250 µg kg^−1^ per week is selected for optimal irisin administration doses.^[^ ^19^, ^20^ ^]^ Exogenous irisin (250 µg kg^−1^ per week) is injected into hypoxic mice. Irisin injection markedly decreases the wall thickness of the pulmonary arteries in hypoxia mice compared with that in normoxia mice (**Figure** **7**A–C). Right ventricular function in hypoxia mice, evaluated in terms of RV/LV+S (Figure 7D) and PAVTI (Figure 7E) are also alleviated following irisin injection. Exogenous injection of irisin also reduces RVSP in hypoxia mice (Figure 7F,G). Furthermore, analysis of the protein expression of PCNA and cell cycle‐related proteins, including cyclinD1, CDK1 and CDK4, reveals that irisin injection significantly reduces the expression of these proliferation and cell cycle‐related proteins (Figure 7H,I). Accordingly, evaluation of the SuHx mouse model yields similar results. Injection of exogenous irisin into SuHx mice effectively improves the wall thickness of pulmonary arteries (Figure 7J–L), RV/LV+S (Figure 7M), PAVTI (Figure 7N), RVSP (Figure 7O,P), and protein expression of PCNA, cyclinD1, CDK1, and CDK4 (Figure 7Q,R). To further verify that the hypoxia model yields identical responses to the SuHx treatment as shown by the experimental groups, the effect size for *t*‐test results, Cohen's d, is calculated and its thresholds interpreted as small (0.2), medium (0.5), and large (0.8) effects.^[^ ^21^ ^]^ The detailed data are presented in Table S8 (Supporting Information). These results further highlight the potential therapeutic value and clinical translational significance of irisin in PAH. The present study clearly demonstrates the model‐to‐model differences in Tables S4,S5, and S9 (Supporting Information). Validation of the overexpression and knockdown efficacy of the relevant genes is presented in Figure S10 (Supporting Information).

## 3 Discussion

**Figure 8:**
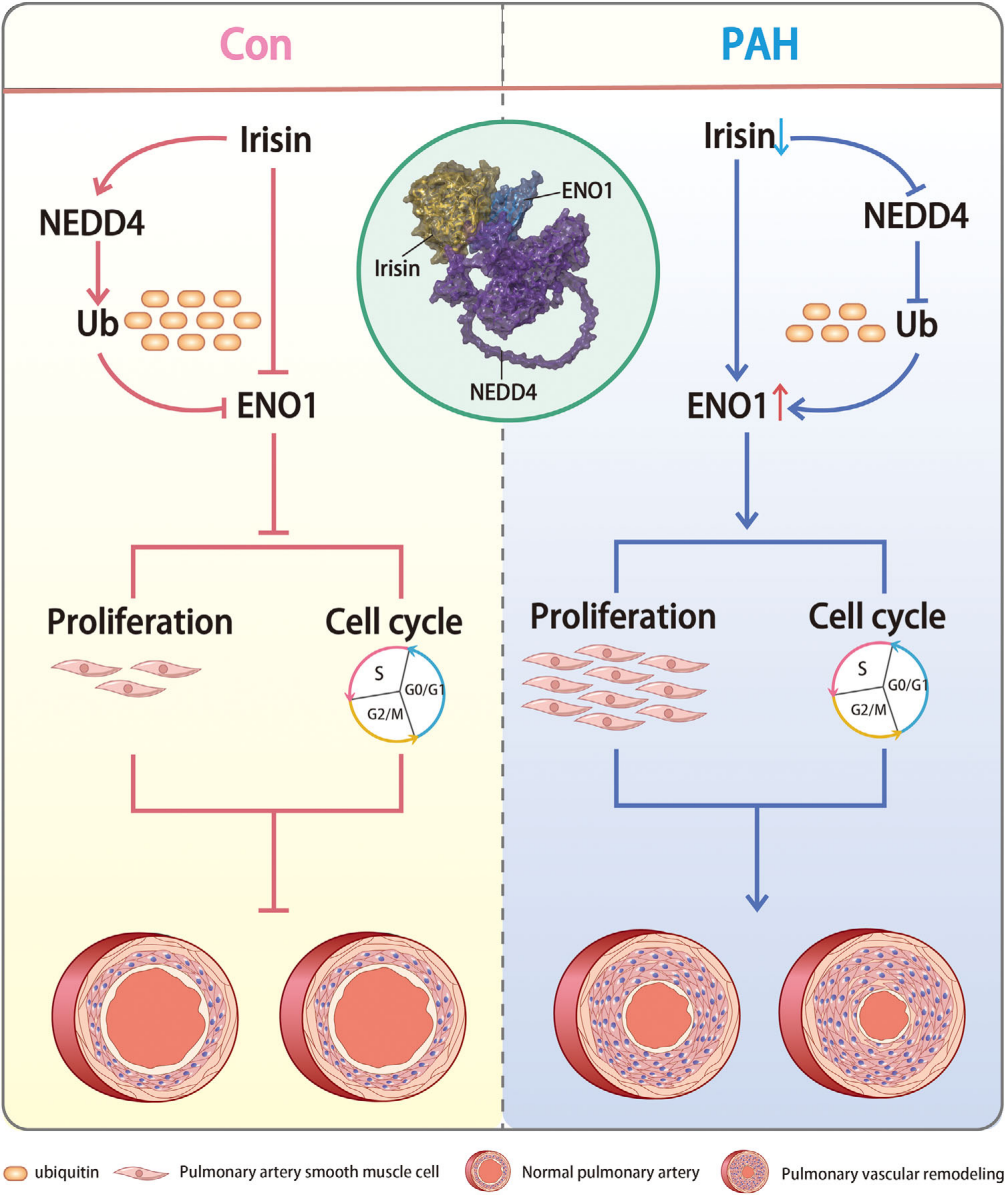
A schematic illustration shows the molecular mechanisms by which irisin alleviates PAH. During normal conditions, irisin recruits E3 ligase NEDD4 to enhance the ubiquitination of its interactive protein ENO1, which stabilizes the expression of ENO1. In PAH condition, the reduced expression of irisin further decreases the recruitment of NEDD4, resulting in the increase of ENO1, which further promotes proliferation and cell cycle progression of PASMCs.

The present study identifies a novel protective role of irisin in PAH pathology. As an exercise‐induced myokine, irisin represents a potentially valuable therapeutic target not only for its anti‐oxidative or anti‐inflammatory functions, but also for its beneficial effects in a variety of chronic diseases such as kidney disease, diabetes, hypertension and others.^[^ ^1^, ^22^, ^23^, ^24^ ^]^ However, the role of irisin in PAH has not been defined. Here, we demonstrate that irisin is markedly reduced in both plasma and pulmonary arteries of patients with PAH, as well as in two different rodent PAH mouse models. Pulmonary vascular expression of irisin is associated with disease severity and prognosis in patients with PAH. It is worth highlighting the negative correlation between irisin and PVR, which further accentuates the potential of irisin as a promising biomarker that reflects pulmonary vascular remodeling in PAH, as PVR is closely associated with right‐heart function and vascular remodeling.^[^ ^25^, ^26^ ^]^ Our in vivo studies demonstrate that PAH development and pulmonary vascular remodeling negatively correlate with irisin expression levels. The schematic illustration showing the molecular mechanisms by which irisin alleviates PAH is presented in **Figure** **8**.

Irisin may exert a potential protective function against PAH pathogenesis. Overexpression of irisin leads to alleviated hemodynamic status, decreased pulmonary vascular remodeling in response to hypoxia and SuHx mouse models by alleviating proliferation and cell cycle progression. Prior investigation has confirmed the role of irisin in inhibiting the proliferation of vascular smooth cells.^[^ ^27^ ^]^ A protective role of irisin in other chronic diseases has also been demonstrated previously.^[^ ^28^, ^29^, ^30^ ^]^ Previous studies described the effect of irisin on vascular smooth muscle cells in chronic kidney disease, irisin protects against vascular calcification by NLRP3‐mediated vascular smooth muscle cells.^[^ ^27^ ^]^ In addition, irisin may also reduce proliferation and inflammation in acute lung injury.^[^ ^31^ ^]^ Furthermore, irisin shows the biomarker and translation treatment effects in various diseases, such as hypertension,^[^ ^24^ ^]^ Sepsis‐associated encephalopathy^[^ ^32^ ^]^ and type 1 diabetes mellitus.^[^ ^33^ ^]^ However, the present study is the first to demonstrate the function of irisin in PAH and PASMCs. Interestingly, we not only observe decreased expression of irisin but also reveal that irisin was localized mainly in the smooth muscle cells of pulmonary arteries, both in patients with PAH and in a mouse PAH model. The major advantages of irisin in our study are that the present study systematically investigates the role and mechanism in PAH, which reflects both the clinical significance and deep molecular mechanism of patients and mouse models. The above findings demonstrate the significant role of PASMCs in the irisin‐mediated regulation of PAH pathology.

Cell proliferation, migration, cell cycle alteration, and phenotypic transformation are the main PASMCs mechanisms underlying pulmonary vascular remodeling in PAH.^[^ ^34^, ^35^, ^36^ ^]^ We identify that reduced irisin expression may negatively correlate with the proliferation of PASMCs. Meanwhile, decreased level of irisin exacerbates the proliferation and cell cycle progression of PDGF‐induced PASMCs; conversely, overexpression of irisin inhibits its proliferative phenotype. Recently, irisin has been reported to exert anti‐proliferative effects in certain chronic diseases such as diabetes and prostate cancer.^[^ ^37^, ^38^ ^]^ In addition, irisin may also exert various effects in different diseases. Irisin attenuates type 1 diabetic cardiomyopathy by anti‐ferroptosis via SIRT1‐mediated deacetylation of p53,^[^ ^39^ ^]^ it may also alleviate vascular calcification by inhibiting SMC osteoblastic transformation and mitochondria dysfunction.^[^ ^40^ ^]^ Furthermore, irisin lowers blood pressure by improving endothelial dysfunction via AMPK‐Akt‐eNOS‐NO pathway in the spontaneously hypertensive rat^[^ ^24^ ^]^ and protects macrophages from oxidized low‐density lipoprotein‐induced apoptosis by inhibiting the endoplasmic reticulum stress pathway.^[^ ^41^ ^]^ In our study, we investigate the effect of irisin on metabolism. Irisin has been reported to improve metabolic disorders by enhancing β‐cell function and insulin secretion in diabetes.^[^ ^42^, ^43^ ^]^ Our results indicate reduced body weight and decreased blood glucose in hypoxia mouse models, whereas overexpression of irisin reverses the above changes to some extent. Taken together, the present study is the first to demonstrate that irisin effectively suppresses the proliferation of PASMCs in PAH and delays cell cycle progression in vitro.

ENO1 is upregulated in various tumors and plays a significant role in promoting tumor proliferation, resistance to apoptosis, migration and metastasis.^[^ ^44^, ^45^ ^]^ Previous studies indicate that ENO1 regulates the malignant phenotype of pulmonary artery smooth muscle cells via the AMPK‐Akt pathway, which serves as a regulator of pathogenic metabolic reprogramming in hypoxia pulmonary hypertension.^[^ ^46^ ^]^ However, the relationship between irisin and ENO1 regulation has rarely been investigated. To further investigate the regulatory mechanism of irisin on PAH, we perform pull‐down assays combined with mass spectrometry analysis to identify ENO1 as a major irisin‐interacting protein. In the present study, we demonstrate a direct interaction between irisin and ENO1. Additionally, we also show that irisin regulates the malignant phenotype of PAH by downregulating ENO1 expression. The specific underlying mechanisms are the focus of our study. We illustrate that irisin downregulates ENO1 at the protein level instead of the mRNA level, which indicates that post‐translational modification may be involved in the process. We demonstrate that MG132‐treated PASMCs with irisin overexpression show markedly increased ENO1 expression. We also reveal that elevated ubiquitination of ENO1 following irisin overexpression, which indicates that irisin may promote the ubiquitination of ENO1 and, in turn, increase ENO1 degradation. To further investigate the involvement of the E3 ligase in this process, we use UbiBrowser, which predicts NEDD4 as the key ligase in the ubiquitination‐mediated regulation of ENO1. Molecular docking analysis was used to evaluate the structure and precise binding sites within the interacting proteins irisin, ENO1, and NEDD4.

The present study is unique in its evaluation of the clinical translational potential of irisin. Injection of exogenous recombinant irisin into SuHx and hypoxia mice alleviates hemodynamic and right‐heart function and reverses pulmonary vascular remodeling. The therapeutic value of irisin has been assessed in Alzheimer's disease and other neurodegenerative disorders^[^ ^47^, ^48^ ^]^; however, the underlying mechanism requires further exploration. Our study systematically elucidates the function and mechanism of irisin in PAH. We demonstrate its potential as a biomarker and therapeutic target in PAH disease progression, which endows irisin with significant clinical translation value.

The present study has several limitations. First, owing to a lack of blood sample collection during the follow‐up period, we failed to evaluate the fluctuation in irisin concentration throughout PAH disease progression. Second, in terms of the literature report and preliminary experiment of irisin concentration gradient, we only confirm 250 µg kg^−1^ per week as the effective injection dose in PAH mice, however, the optimal therapeutic dose of irisin in patients with PAH remains unknown and needs to be further investigated. In addition, besides smoking, we fail to collect other lifestyle parameters like exercise habits and diet, which limit the comprehensive evaluation of the impact of these factors on irisin levels. Furthermore, the interaction sites among irisin, ENO1, and NEDD4 are predicted by computer molecular docking simulations. However, it still needs to be investigated the role of irisin in molecular interaction and the specific domains involved in mediating ENO1 ubiquitination. Finally, due to the limitations in the detection module of the echocardiography machine and the lack of experimental conditions, we failed to evaluate the fractional area change of mice and are unable to evaluate the effect of irisin on vascular adaptive response.

In conclusion, irisin is a novel protective factor against PAH development and is associated with disease severity and prognosis. Enhanced irisin expression may effectively suppress pulmonary vascular proliferation and remodeling by promoting E3 ligase NEDD4‐mediated ubiquitination and degradation of the interacting protein ENO1. Our study also demonstrates the therapeutic potential of irisin in PAH, emphasizing its significant clinical translational value.

## 4 Experimental Section

### Patient Population and Human Lung Sample

A total of 93 consecutive adult patients with PAH were recruited at Second Affiliated Hospital of Harbin Medical University from January 11, 2018 to December 21, 2021. Also, 50 age‐ and sex‐matched control subjects participated in the present study. Patients enrolled were satisfied with the following criteria: ages between 18 and 75 years old; patients were diagnosed with PAH by right heart catheterization according to the updated WHO clinical classification guidelines.^[^ ^49^ ^]^ Patients with chronic kidney disease or any other disease that may affect the abnormal distribution of irisin were excluded from the study.

The mean follow‐up time was 2.9±1.5 years for recruited patients. The primary endpoint was time to the first clinical worsening event, including all‐cause mortality, re‐hospitalization for a clinical adverse condition related to PAH and lung transplantation. The clinical worsening condition, as previously described, including deteriorating cardiac function and any other PAH‐related worsening that requires hospitalization.^[^ ^13^ ^]^ The above follow‐up information was carefully recorded by review of medical records or follow‐up phone calls.

All the human pulmonary artery samples were obtained from patients with PAH who underwent Heart and/or Lung transplantations. The connective tissue and fat around the pulmonary artery were removed gently. PAH was confirmed using right‐heart catheterization before targeted drug therapy. Human pulmonary artery samples were collected in accordance with the Declaration of Helsinki. Informed consent was obtained from all the patients.

### Measurement of Plasma Irisin Concentration

Blood samples measured for plasma irisin levels were collected from the peripheral veins of the recruited patients, using ethylene diamine tetraacetic acid (EDTA) as an anticoagulant. Blood samples were centrifuged at 1800 g at 4 °C for 15 min. Samples were stored at −80 °C and repeated freeze‐thaw cycles were avoided. Plasma irisin concentration was determined using a competitive ELISA Kit (AdipoGen, Liestal, Switzerland) according to the manufacturer's instructions. Two replicate wells were used for each plasma sample to calculate the mean value.^[^ ^50^ ^]^

### Hemodynamic Measurement

For all recruited patients, right heart catheterization was performed using the Swan–Gans catheter. Baseline hemodynamic variables were measured for all patients, including mPAP, diastolic pulmonary arterial pressure (dPAP), systolic pulmonary arterial pressure (sPAP), mean right ventricular pressure (mRVP), diastolic right ventricular pressure (dRVP), systolic right ventricular pressure (sRVP), mean right atrial pressure (mRAP), diastolic right atrial pressure (dRAP), systolic right atrial pressure (sRAP), cardiac output (CO), cardiac index (CI) and pulmonary vascular resistance (PVR). CO was calculated in triplicate by the thermo‐dilution technique with ice‐cold isotonic saline solution. PVR was assessed using the equation PVR = PAMP − PCWP (trans‐pulmonary gradient) divided by CO.

### Animal Studies, Hemodynamics, Echocardiography, and Histology

All animal experiments were approved by the Second Affiliated Hospital of Harbin Medical University (Harbin, China). Two PAH animal models were used in the present study: a hypoxia mouse model and a SU5416/hypoxia (SuHx, Sigma, 204005‐46‐9, Germany) mouse model. Animals were randomized into different experimental groups. For the hypoxia mouse model, adult male mice (8 weeks old) were maintained under 10% oxygen (hypoxia) for four weeks (12‐h light/dark cycle).^[^ ^51^ ^]^ For the SuHx mouse model, adult mice (8 weeks old) were injected with SU5416 (20 mg kg^−1^, Sigma, 204005‐46‐9, Germany), once a week, and maintained under 10% oxygen (hypoxic) for three weeks.^[^ ^52^ ^]^

The adeno‐associated virus 5 (AAV5) vectors carrying targeted sequences were purchased from Hanheng Biotech (Shanghai, China). The inhaled AAV5 vectors are effectively delivered to the lungs and pulmonary arteries. The pHBAAV‐CMV‐MCS‐3flag construct was used for irisin overexpression. The hemodynamic parameter RVSP was measured using standardized protocols. The Fulton index was calculated as the ratio of the right ventricular weight to the left ventricle and septum (RV/LV+S) weight. Right ventricular function was evaluated using echocardiographic measurements of the pulmonary artery velocity time integral (PAVTI). To further investigate the remodeling of the pulmonary vessel wall, formaldehyde‐fixed and paraffin‐embedded lung tissue sections of mice were stained with hematoxylin and eosin and subsequently observed under a microscope to calculate the wall thickness of the pulmonary arteries. All the pulmonary arteries used in the experiments were the arteries with 50–100 µm in diameter and ≈15 arteries were analyzed in each mouse. The degree of medial pulmonary artery wall thickness was investigated by Image J and analyzed as previously described: medial wall thickness = (total vascular area‐lumen area)/total vascular area.^[^ ^53^ ^]^

To further investigate the clinical treatment potential of irisin, hypoxia and SuHx mouse models were injected with recombinant irisin at 250 µg kg^−1^ per week (Phoenix Pharmaceuticals, 067‐20A, German). Irisin was injected subcutaneously into the mouse in the abdomen. Hemodynamics, echocardiography, and pulmonary vascular remodeling were evaluated in mice injected with irisin. The animal study was approved by the ethics committee of the Second Affiliated Hospital of Harbin Medical University (#GZR2023‐03).

### Cell Culture and Treatments

Primary human PASMCs were purchased from ScienCell (Shanghai, China). The primary mouse PASMCs were purchased from iCell (Shanghai, China). All cell cultures were maintained at 37 °C in 5% CO2 and 95% air. PDGF (R&D Systems, 220‐BB‐50, USA) was used to induce the proliferation of PASMCs. Primary cells from passages 5 to 9 were used in the experiments.

Human PASMCs were treated with PDGF‐BB at a concentration of 50 ng mL^−1^ for 24 or 48 h. In addition, PASMCs were also treated with the following reagents: recombinant irisin (50, 100, or 200 ng mL^−1^; Phoenix Pharmaceuticals, 067‐20A, German) and MG132 (20 µM; S2619, Selleck, Houston, Texas, USA).

To overexpress irisin in PASMCs, an irisin‐overexpressing vector (GeneCreate, Wuhan, China) was transfected into the cells. To knockdown irisin expression, PASMCs were transfected with a siRNA targeting irisin (GenePharme, Wuhan, China). Targeted interference sequences are listed in Table S7 (Supporting Information). These transfected PASMCs were used in the subsequent experiments.

### Quantitative Real‐Time Polymerase Chain Reaction (qPCR)

qPCR was conducted as previously described.^[^ ^54^ ^]^ Total RNA was isolated from PASMCs and tissues using TRIzol reagent (Invitrogen, USA). Purified RNA was reverse transcribed to cDNA using a Transcriptor First Strand cDNA Synthesis Kit (Roche, Germany) according to the manufacturer's protocol. qPCR was performed using SYBR® Green (Roche, Germany) on a qPCR Detection System (CFX Connect, Bio‐Rad, USA). Detailed information on the primers used for qPCR is presented in Table S6 (Supporting Information). The quantifications were conducted using the 2‐Δ (Δ Ct) method that was described previously.^[^ ^55^ ^]^

### Western Blotting

Cells and tissues were lysed in radio immunoprecipitation assay (RIPA buffer; Beyotime, China) containing a protease inhibitor (Roche, Basel, Switzerland) and a phosphatase inhibitor cocktail (Bimake, B15001, USA). The lysates were centrifuged at 12000 g for 15 min, and the supernatant was collected. The lysates were boiled for 5 min in sodium dodecyl sulfate (SDS) loading buffer (Beyotime, P0015L, China), resolved by SDS‐polyacrylamide gel electrophoresis, transferred to polyvinylidene fluoride (PVDF) membranes (Roche, Germany), and the nonspecific sites were blocked with milk. The membranes were incubated with primary antibodies (as indicated) overnight at 4 °C. Protein signals were detected using an enhanced chemiluminescence substrate and horseradish peroxidase (HRP)‐conjugated secondary antibodies. β‐actin served as the internal control. The Western Blot band intensity quantification analysis is normalized using loading controls. The antibodies used for western blotting are listed in Table S8 (Supporting Information).

### Cell Cycle Analysis

Cell cycle was assessed using the Cell Cycle and Apoptosis Analysis Kit (Beyotime, C1052, China) and flow cytometry. Prior to proliferation assays, cells were synchronized in G0/G1 phase via serum deprivation (0.1% FBS for 24 h). After treatment, the PASMCs were collected, centrifuged at 1000 g for 5 min, and the supernatant was discarded. Cells were fixed in chilled 70% ethanol (1 mL) at 4 °C for 30 min and then stained with propidium iodide solution (0.5 mL). Finally, flow cytometry was used to analyze the cells at an excitation wavelength of 488 nm.

### Co‐Immunoprecipitation (Co‐IP) Assay

Protein samples were incubated with the IP antibodies against ENO1 (1:50, Abclonal, Wuhan, China), NEDD4 (1:50, Proteintech, Wuhan, China), FLAG (1:50, Abclonal, Wuhan, China), and IgG (1:50, Abcam, UK) overnight at 4 °C on a rotating wheel. Protein A/G beads (Bimake, B23202, USA) were washed thrice with RIPA buffer and then blocked with 3% bovine serum albumin (BSA) at 4 °C for 30 min. The protein lysate‐antibody mixtures were coupled to protein A/G beads by incubating for 6 h at 4 °C. After discarding the supernatant, bound proteins were eluted by adding 100 µL of lysis buffer (1×SDS loading buffer) to the beads. The samples were boiled for 5 min and separated by SDS‐PAGE.

### Immunofluorescence Staining

For immunofluorescence staining, formaldehyde‐fixed paraffin‐embedded human and mouse tissues were rehydrated and deparaffinized. Cells were fixed with 4% paraformaldehyde at room temperature for 20 min and then incubated with PBS/0.4% Triton X100/1% BSA for 30 min. The tissues and cells were incubated with primary antibodies against irisin (1:100; Abclonal, Wuhan, China), SMA (1:100; Boster, Beijing, China), ENO1 (1:100; Abclonal, Wuhan, China), and Ki67 (1:100; Abclonal, Wuhan, China) at 4 °C overnight. The tissues and cells were washed three times with PBS and incubated with secondary antibodies at room temperature for 1–2 h. Cells were stained with 4ʹ,6‐diamidino‐2‐phenylindole (DAPI) for 10 min at room temperature. All the pulmonary arteries used in the experiments were the arteries with diameters less than 100 µm. High‐quality images were obtained using a confocal microscopy (ZEISS LSM800, Germany).

### Pull‐Down and Mass Spectrometry Analyses

The pull‐down assay was performed in two steps. First, His pull‐down assay was performed by mixing equal amounts (0.5 mg) of His‐irisin and FLAG‐ENO1 fusion proteins (synthesized by GeneCreate Biological Engineering, Wuhan, China) on ice for 3 h. Then, the mixture was loaded onto a Glutathione Sepharose 4B resin column. After washing five times with the wash buffer, the proteins were eluted with wash buffer supplemented with 15 mm reduced glutathione. The eluates were separated by 12% SDS‐PAGE, transferred to PVDF membranes (Millipore, Billerica, MA, USA), and probed with anti‐His (Sigma‐Aldrich, Germany) and anti‐FLAG antibodies (Abclonal, Wuhan, China) to evaluate the direct interaction between irisin and ENO1. His and FLAG proteins (GeneCreate, Wuhan, China) were used as negative controls.

For the second part of the pull‐down assay, His‐irisin fusion proteins were incubated with human PASMCs, the mixture was processed as described above, and the proteins were separated by SDS‐PAGE. In addition, spectrometric analysis was performed to identify protein that interacts with irisin.

### Cell Counting Kit‐8 Assay

PASMCs proliferation was assessed using a Cell Counting Kit (CCK8, Dojindo, CK04‐500T, China) according to the manufacturer's instructions. After treatment, cell growth was assessed in 96‐well plates by adding 10 µL of CCK8 reagent to each well. The plates were then placed in an incubator for 2–4 h in the dark. The absorbance was measured at 450 nm using an ELISA assay reader, and the cell proliferation rate was calculated.

### Computational Modeling and Molecular Docking Assay

First, the ligand protein was rotated 70 000 times relative to the receptor protein in multiple directions (maximum number of rotations), and the direction of each ligand protein was screened to determine the optimal docking score for the receptor protein. The highest 1000 rotations were clustered using the root mean square distance between the matching atoms in each pair of rotating structures. The selected structure in each cluster was the structure with the most neighbors in the cluster. After docking was completed, by default, the side chains of residues within a range of 5 Å near the interaction interface between the two proteins were optimized to minimize the conflicts and optimize interactions.

### Statistical Analyses

Statistical analyses were performed using SPSS‐PASW 26.0 (IBM) or GraphPad Prism 7 software. Mean ± standard deviation was expressed for normally distributed variables. Data that were not normally distributed are expressed as percentages, numbers, and medians with corresponding interquartile ranges. The clinical features, demographics, and hemodynamic parameter subgroups by median levels of irisin using the *t*‐test, Chi‐square test, or Mann–Whitney U test as appropriate were compared.

For the correlation analyses, a scatter plot was created for continuous variables. The Pearson correlation analysis method was applied to analyze the linear correlation of variables; otherwise, for categorical or abnormally distributed continuous variables, Spearman's rank correlation method was used.

Two‐step survival analysis was used to evaluate the prognostic significance and independent correlation between plasma irisin level and mortality.^[^ ^56^ ^]^ First, univariate Cox proportional intervals were used to assess HRs and 95% CIs for the association between covariates and outcomes. Second, a multivariate Cox regression model was used to evaluate HRs and 95% CIs for the association between plasma irisin levels and outcomes that were adjusted for variables that were meaningful in the univariate analysis. The log‐rank test was used to compare survival among the different patient groups. Statistical significance was set at *p* < 0.05.

### Study Approval

Handling of mice and all the experiments were conducted in accordance with local laws for animal protection. All enrolled subjects gave their written informed consent. All the human pulmonary artery samples were obtained from patients with PAH undergone Heart or Lung transplantations. Human pulmonary artery samples were collected in accordance with the Declaration of Helsinki. Informed consent was obtained from all the patients. The study was approved by the ethics committee of the Second Affiliated Hospital of Harbin Medical University (#GZR2023‐03).

## Conflict of Interest

The authors declare no conflict of interest.

## Supporting information



Supporting Information

## Data Availability

The data that support the findings of this study are available from the corresponding author upon reasonable request.
