# Supplementary material for: Irisin Attenuates Pulmonary Vascular Remodeling in Pulmonary Arterial Hypertension via Ubiquitin‐Mediated Regulation of ENO1
Source: Adv Sci (Weinh). 2025 Jun 25;12(35):e00096. doi: 10.1002/advs.202500096 (PMC12463039; doi:10.1002/advs.202500096)

**Online data supplement**

**Supplemental Tables**

**Supplemental Figures**

**Supplemental Tables**

**Table S1.** Baseline Characteristics of study population in relation to plasma irisin level.

|  | All patients  (n=93) | High-Irisin  (n=46) | Low-Irisin  (n=47) | *P* Value^*^ |
| --- | --- | --- | --- | --- |
| Age, years | 47.7±18.0 | 46.3±18.2 | 49.1±19.8 | 0.465 |
| Female gender, n (%) | 68(73.1%) | 33(71.7%) | 35(74.5%) | 0.768 |
| HR^a)^, bpm | 89.0±15.1 | 89.6±13.0 | 88.4±17.1 | 0.708 |
| SBP^b)^, mmHg | 120.6±20.1 | 120.6±16.6 | 120.5±23.2 | 0.990 |
| RVD^c)^, mm | 29.1±8.7 | 29.6±8.1 | 28.7±9.2 | 0.636 |
| TnI^d)^, μg/ml | 0.02(0, 0.05) | 0.01(0, 0.06) | 0.02(0,0.04) | 0.916 |
| NT-proBNP^e)^, pg/ml | 1520.0(699.5, 4710.0) | 838.0(326.3, 1170.5) | 2163.0(1432.0, 5218.0) | <0.001 |
| WHO^f)^ functional class, n(%) |  |  |  | 0.023 |
| II | 35(37.6%) | 22(47.8%) | 13(27.7) |  |
| III | 44(47.3%) | 20(43.5%) | 24(51.1) |  |
| IV | 14(15.1%) | 4(8.7%) | 10(21.3%) |  |
| CHOL^g)^, mmol/L | 3.9±1.3 | 4.2±1.4 | 3.7±1.1 | 0.086 |
| LDL-C^h)^, mmol/L | 2.4±1.0 | 2.6±1.1 | 2.3±0.9 | 0.116 |
| TG^i)^, mmol/L | 1.4±0.8 | 1.6±1.0 | 1.2±0.4 | 0.019 |
| HDL-C^j)^, mmol/L | 1.1±0.4 | 1.1±0.4 | 1.0±0.4 | 0.312 |
| UA^k)^, μmol/L | 442.2±163.5 | 421.3±154.6 | 462.6±171.3 | 0.225 |
| Irisin, μg/ml | 3.0±0.6 | 3.4±0.3 | 2.6±0.5 | <0.001 |
| mPAP^l)^, mmHg | 51.2±20.8 | 46.3±20.3 | 56.0±20.4 | 0.024 |
| sPAP^m)^, mmHg | 74.7±29.1 | 68.2±30.4 | 81.2±26.5 | 0.030 |
| dPAP^n)^, mmHg | 37.2±17.4 | 33.5±15.4 | 40.8±18.6 | 0.044 |
| mRVP^o)^, mmHg | 33.1±14.1 | 30.0±14.1 | 36.3±13.6 | 0.037 |
| sRVP^p)^, mmHg | 73.0±29.2 | 67.7±31.3 | 78.3±26.2 | 0.087 |
| dRVP^q)^, mmHg | 7.0(4.0, 12.8) | 6.0(4.0, 11.5) | 8.5(3.0, 13.0) | 0.330 |
| mRAP^r)^, mmHg | 9.0(5.0, 12.0) | 8.0(5.3, 11.0) | 9.0(5.0, 16.0) | 0.636 |
| sRAP^s)^, mmHg | 13.6±10.6 | 12.2±5.1 | 14.9±13.7 | 0.208 |
| dRAP^t)^, mmHg | 6.0(3.0±8.3) | 12.2±5.1 | 14.9±13.7 | 0.881 |
| CO^u)^, L·min^-1^ | 4.3±1.8 | 4.4±1.8 | 4.2±1.8 | 0.442 |
| CI^v)^, L·min^-1^·m^-2^ | 2.2(1.8, 3.4) | 2.2(1.8, 3.6) | 2.3(1.5, 3.3) | 0.282 |
| PVR^w)^, Woods units | 8.7(5.5, 17.3) | 8.1(5.6, 14.4) | 11.2(5.4, 19.6) | 0.144 |

^a)^HR: heart rate; ^b)^SBP: systolic blood pressure; ^c)^RVD: right ventricular diameter; ^d)^TnI: troponin I; ^e)^NT-proBNP: N-terminal pro-brain natriuretic peptide; ^f)^WHO: World Health Organization; ^g)^CHOL: total cholesterol; ^h)^LDL-C: low-density lipoprotein cholesterol; ^i)^TG: triglycerides; ^j)^HDL-C: high-

density lipoprotein cholesterol; ^k)^UA: uric acid; ^l)^mPAP: mean pulmonary arterial pressure; ^m)^sPAP: systolic pulmonary arterial pressure; ^n)^dPAP: diastolic pulmonary arterial pressure; ^o)^mRVP: mean right ventricular pressure; ^p)^sRVP: systolic right ventricular pressure; ^q)^dRVP: diastolic right ventricular pressure; ^r)^mRAP: mean right atrial pressure; ^s)^sRAP: systolic right atrial pressure; ^t)^dRAP: diastolic right atrial pressure; ^u)^CO: cardiac output; ^v)^CI: cardiac index; ^w)^PVR: pulmonary vascular resistance.

^*^comparison between High-Irisin group and Low-Irisin group.

**Table S2.** Correlation of plasma irisin levels with clinical and hemodynamic parameters in IPAH patients.

| Parameters | R Value | *P* Value |
| --- | --- | --- |
| NT-proBNP^a)^, pg/ml | -0.503 | <0.001 |
| TG^b)^, mmol/L | 0.302 | 0.005 |
| mPAP^c)^, mmHg | -0.316 | 0.002 |
| sPAP^d)^, mmHg | -0.330 | 0.001 |
| dPAP^e)^, mmHg | -0.276 | 0.007 |
| mRVP^f)^, mmHg | -0.308 | 0.004 |
| CO^g)^, L·min^-1^ | 0.131 | 0.215 |
| CI^h)^, L·min^-1^·m^-2^ | 0.245 | 0.030 |
| PVR^i)^, Woods units | -0.266 | 0.013 |
| 6MWD^j)^, m | 0.258 | 0.012 |
| Hypertension,n(%) | -0.090 | 0.393 |
| Diabetes,n(%) | -0.118 | 0.269 |
| Tumor,n(%) | -0.039 | 0.708 |
| Smoking,n(%) | 0.012 | 0.908 |
| WHO^k)^ functional class,n(%) | -0.236 | 0.023 |

^a)^NT-proBNP: N-terminal pro-brain natriuretic peptide ; ^b)^TG: triglycerides; ^c)^mPAP: mean pulmonary arterial pressure; ^d)^sPAP: systolic pulmonary arterial pressure; ^e)^dPAP: diastolic pulmonary arterial pressure; ^f)^mRVP: mean right ventricular pressure; ^g)^CO: cardiac output; ^h)^CI: cardiac index; ^i)^PVR: pulmonary vascular resistance; ^j)^6MWD:6 minute walk distance; ^k)^WHO : World Health Organization.

**Table S3.** Univariate and Multivariate analysis of risk factors in patients with IPAH.

| Variables | Univariate analysis | | Mutivariate analysis | |
| --- | --- | --- | --- | --- |
|  | HR^a)^[95%CI^b]^] | *P* Value | HR[95%CI] | *P* Value |
| Age, years | 0.999(0.977, 1.022) | 0.933 | - | - |
| Sex, n(%) | 0.631(0.272, 1.462) | 0.282 | - | - |
| Irisin, μg/ml | 0.499(0.293, 0.852) | 0.011 | 0.479(0.277, 0.830) | 0.009 |
| WHO^c)^ functional class, n(%) | 1.477 (0.846, 2.576) | 0.170 | - | - |
| NT-proBNP^d)^, pg/ml | 1.755(0.956, 2.134) | 0.292 | - | - |
| mPAP^e)^, mmHg | 1.030 (0.896. 1.144) | 0.101 | - | - |
| CI^f)^, L·min^-1^·m^-2^ | 0.555(0.308, 0.999) | 0.050 | - | - |
| PVR^g)^, Woods units | 1.067(1.027, 1.108) | 0.001 | 1.057(1.015, 1.100) | 0.007 |

^a)^HR:hazard ratio; ^b)^CI: confidence interval; ^c)^WHO: World Health Organization; ^d)^NT-proBNP: N-terminal pro-brain natriuretic peptide; ^e)^mPAP: mean pulmonary arterial pressure; ^f)^CI: cardiac index; ^g)^PVR: pulmonary vascular resistance.

**Table S4.** Comparison of hemodynamic parameters in mouse model.

| Parameters | Hypoxia mouse model | | |  | SuHx mouse model | | |
| --- | --- | --- | --- | --- | --- | --- | --- |
|  | Con | HYP^a)^ | *P* value |  | Con | SuHx | *P* value |
| RVSP^b)^,mmHg | 22.03±3.41 | 28.30±2.72 | 0.005 |  | 20.37±0.96 | 37.78±4.65 | <0.001 |
| RV/LV+S^c)^ | 0.34±0.14 | 1.02±0.19 | <0.001 |  | 0.45±0.12 | 0.89±0.11 | <0.001 |
| PAVTI,mm^d)^ | 38.71±1.91 | 34.51±1.44 | 0.002 |  | 39.68±1.39 | 32.53±4.71 | 0.005 |

^a)^HYP: hypoxia; ^b)^RVSP: right ventricular systolic pressure; ^c)^RV/LV+S: right ventricular to left ventricular + septum; ^d)^PAVTI: pulmonary artery velocity time integral.

**Table S5.** Comparable data presentation of AAV-irisin overexpression effect among hypoxia and SuHx mouse model.

| Parameters | Hypoxia mouse model | | | |  | SuHx mouse model | | | |
| --- | --- | --- | --- | --- | --- | --- | --- | --- | --- |
|  | AAV^a)^-null | AAV-irisin | AAV-null+hypoxia | AAV-irisin+hypoxia |  | AAV-null | AAV-irisin | AAV-null+SuHx | AAV-irisin+SuHx |
| RVSP^b)^,mmHg | 20.73±2.65 | 17.21±2.46 | 40.16±1.12 | 21.75±3.01 |  | 20.12±2.30 | 15.97±3.25 | 39.38±5.47 | 27.79±2.45 |
| RV/LV+S^c)^ | 0.45±0.11 | 0.47±0.13 | 0.93±0.09 | 0.59±0.14 |  | 0.42±0.06 | 0.45±0.07 | 0.95±0.06 | 0.62±0.08 |
| PAVTI^d)^,mm | 32.28±2.97 | 40.69±1.28 | 33.97±1.92 | 38.21±1.61 |  | 37.12±1.63 | 41.37±4.10 | 31.53±5.22 | 40.10±2.08 |
| Pulmonary artery thickness/diameter, % | 0.14±0.05 | 0.08±0.02 | 0.37±0.09 | 0.11±0.04 |  | 0.14±0.04 | 0.08±0.04 | 0.30±0.13 | 0.14±0.05 |
| Ki67 Fluorescence/Vessel area, RUF/pixels^2^ | 8.66±3.07 | 9.42±6.18 | 55.52±16.55 | 10.89±5.34 |  | 12.84±4.03 | 10.25±7.12 | 43.33±15.71 | 9.89±4.50 |

^a)^AAV: adeno-associated virus; RVSP ^b)^: right ventricular systolic pressure; ^c)^RV/LV+S: right ventricular to left ventricular + septum; ^d)^PAVTI: pulmonary artery velocity time integral.

**Table S6.** Molecular docking detail of irisin and ENO1.

| Set1 Residues | Set2 Residues | Distance(A) | Specific Interactions | #HB | #Salt Bridges | #Pi Stacking | #Disulfides | #vdW Clash |
| --- | --- | --- | --- | --- | --- | --- | --- | --- |
| A:Arg 399 | B:Gln 78 | 1.9 | 2x hb to B:Gln 78 | 2 | 0 | 0 | 0 | 0 |
| A:Glu 9 | B:Leu 74 | 2.3 | 1x hb to B:Leu 74 | 1 | 0 | 0 | 0 | 0 |
| A:Glu 401 | B:Gln 78 | 1.6 | 1x hb to B:Gln 78 | 1 | 0 | 0 | 0 | 0 |
| A:Asn 407 | B:Gln 108 | 2.3 | 1x hb to B:Gln 108 | 1 | 0 | 0 | 0 | 0 |
| A:Ser 400 | B:Gln 103 | 1.8 | 1x hb to B:Gln 103 | 1 | 0 | 0 | 0 | 0 |
| A:Thr 204 | B:Asn 81 | 2.4 | 1x hb to B:Asn 81 | 1 | 0 | 0 | 0 | 0 |
| A:Lys 192 | B:Asn 81 | 1.9 | 1x hb to B:Asn 81 | 1 | 0 | 0 | 0 | 0 |
| A:Tyr 188 | B:Asn 81 | 2 | 1x hb to B:Asn 81 | 1 | 0 | 0 | 0 | 0 |

**Table S7.** Molecular docking detail of irisin, ENO1 and NEDD4.

| Set1 Residues | Set2 Residues | Distance(A) | Specific Interactions | #HB | #Salt Bridges | #Pi Stacking | #Disulfides | #vdW Clash |
| --- | --- | --- | --- | --- | --- | --- | --- | --- |
| A:Leu 29 | C:Phe 909 | 1.2 | 7x clash to C:Phe 909 | 0 | 0 | 0 | 0 | 7 |
| A:Gly 417 | C:Leu 879 | 1.2 | 7x clash to C:Leu 879 | 0 | 0 | 0 | 0 | 7 |
| A:Phe 30 | C:Thr 904 | 1.4 | 6x clash to C:Thr 904 | 0 | 0 | 0 | 0 | 6 |
| A:Ser 418 | C:Leu 879 | 1.6 | 5x clash to C:Leu 879 | 0 | 0 | 0 | 0 | 5 |
| B:Glu 55 | C:Phe 947 | 1.4 | 4x clash to C:Phe 947 | 0 | 0 | 0 | 0 | 4 |
| B:Leu 54 | C:Phe 947 | 1.3 | 3x clash to C:Phe 947 | 0 | 0 | 0 | 0 | 3 |
| B:Leu 54 | C:Ile 1186 | 1.7 | 3x clash to C:Ile 1186 | 0 | 0 | 0 | 0 | 3 |
| A:Phe 24 | C:Arg 901 | 1.3 | 3x clash to C:Arg 901 | 0 | 0 | 0 | 0 | 3 |
| A:Leu 410 | C:His 878 | 1.7 | 2x clash to C:His 878 | 0 | 0 | 0 | 0 | 2 |
| A:Gly 417 | C:Arg 880 | 1.6 | 2x clash to C:Arg 880 | 0 | 0 | 0 | 0 | 2 |
| A:Arg 178 | C:His 878 | 0.4 | 27x clash to C:His 878 | 0 | 0 | 0 | 0 | 27 |
| B:Glu 57 | C:Lys 951 | 2.1 | 1x salt bridge to C:Lys 951 | 0 | 1 | 0 | 0 | 0 |
| A:Lys 27 | C:Thr 902 | 1.4 | 1x hb to C:Thr 902 | 1 | 0 | 0 | 0 | 0 |
| A:Val 123 | C:Thr 902 | 1.5 | 1x clash to C:Thr 902 | 0 | 0 | 0 | 0 | 1 |
| A:Arg 178 | C:Pro 876 | 1.6 | 1x clash to C:Pro 876 | 0 | 0 | 0 | 0 | 1 |
| A:Arg 178 | C:Leu 879 | 1.6 | 1x clash to C:Leu 879 | 0 | 0 | 0 | 0 | 1 |
| A:Glu 413 | C:His 878 | 1.5 | 1x clash to C:His 878 | 0 | 0 | 0 | 0 | 1 |
| A:Glu 415 | C:Gly 881 | 1.6 | 1x clash to C:Gly 881 | 0 | 0 | 0 | 0 | 1 |
| A:Lys 119 | C:Thr 904 | 0.9 | 16x clash to C:Thr 904 | 0 | 0 | 0 | 0 | 16 |
| A:Gly 28 | C:Arg 901 | 0.6 | 14x clash to C:Arg 901 | 0 | 0 | 0 | 0 | 14 |

**Table S8.** The effects of Irisin in the SuHx treatment and hypoxia models.

| Variable | Group | Number | | Mean± SD^a)^ | Estimated means difference (95% CI^b)^) | *p* value | Cohen’s *d*（95% CI） |
| --- | --- | --- | --- | --- | --- | --- | --- |
| Thickness/diameter (%) | HYP^c)^ | | 6 | 0.359±0.165 | -0.163(-0.317, -0.080) | 0.041 | 1.354(0.054,1.602) |
|  | HYP+Irisin | | 6 | 0.196±0.040 |  |  |  |
| Thickness/diameter (%) | SU5416 | | 6 | 0.340±0.148 | -0.213(-0.370; -0.056) | 0.015 | 1.87(0.449,3.231) |
|  | SU5416+Irisin | | 6 | 0.127±0.063 |  |  |  |
| RV/LV+S^d)^ | HYP | | 6 | 0.810±0.142 | -0.314(-0.464, -0.164) | <0.001 | 2.688(1.035,4.278) |
|  | HYP+Irisin | | 6 | 0.495±0.083 |  |  |  |
| RV/LV+S | SU5416 | | 6 | 0.865±0.139 | -0.419(-0.556, -0.281) | <0.001 | 3.912(1.85,5.915) |
|  | SU5416+Irisin | | 6 | 0.446±0.060 |  |  |  |
| PAVTI^e)^ (mm) | HYP | | 6 | 34.381±1.243 | 5.255(3.251,7.256) | <0.001 | 3.373(1.498,5.188) |
|  | HYP+Irisin | | 6 | 39.636±1.818 |  |  |  |
| PAVTI (mm) | SU5416 | | 6 | 30.163±3.712 | 7.950(4.005,11.900) | <0.001 | 2.592(0.968,4.153) |
|  | SU5416+Irisin | | 6 | 38.113±2.242 |  |  |  |
| RVSP^f)^ (mmHg) | HYP | | 6 | 36.756±5.35 | -10.136(-15.401, -4.871) | 0.002 | 2.477(0.887,4.002) |
|  | HYP+Irisin | | 6 | 26.620±2.200 |  |  |  |
| RVSP (mmHg) | SU5416 | | 6 | 39.426±3.871 | -15.987(-20.546, -11.429) | <0.001 | 4.512(2.234,6.737) |
|  | SU5416+Irisin | | 6 | 23.438±3.182 |  |  |  |
| PCNA^g)^ | HYP | | 3 | 1.187±0.294 | -0.830(-1.615, -0.0411) | 0.044 | 2.385(0.065,4.579) |
|  | HYP+Irisin | | 3 | 0.556±0.393 |  |  |  |
| PCNA | SU5416 | | 3 | 1.198±0.188 | -0.520(-1.017, -0.021) | 0.047 | 2.311(0.024,4.47) |
|  | SU5416+Irisin | | 3 | 0.685±0.251 |  |  |  |
| CyclinD1 | HYP | | 3 | 2.008±0.432 | -1.094(-1.844, -0.343) | 0.016 | 3.302(0.531,5.969) |
|  | HYP+Irisin | | 3 | 0.914±0.182 |  |  |  |
| CyclinD1 | SU5416 | | 3 | 1.717±0.075 | -1.305(-1.566, -1.043) | <0.001 | 11.328(3.732,19.05) |
|  | SU5416+Irisin | | 3 | 0.420±0.145 |  |  |  |
| CDK^h)^1 | HYP | | 3 | 1.238±0.193 | -0.614(-1.145, -0.084) | 0.032 | 2.625(0.191,4.935) |
|  | HYP+Irisin | | 3 | 0.624±0.269 |  |  |  |
| CDK1 | SU5416 | | 3 | 1.585±0.442 | -0.994(-1.774, -0.214) | 0.024 | 2.89(0.327,5.336) |
|  | SU5416+Irisin | | 3 | 0.591±0.202 |  |  |  |
| CDK4 | HYP | | 3 | 1.296±0.051 | -0.444(-0.592, -0.300) | 0.001 | 6.811(2.03,11.603) |
|  | HYP+Irisin | | 3 | 0.851±0.077 |  |  |  |
| CDK4 | SU5416 | | 3 | 1.387±0.069 | -0.615(-1.044, -0.187) | 0.016 | 3.254(0.507,5.894) |
|  | SU5416+Irisin | | 3 | 0.772±0.258 |  |  |  |

^a)^SD: standard deviation; ^b)^CI: confidence interval; ^c)^HYP: hypoxia; ^d)^ RV/LV+S: right ventricular to left ventricular + septum; ^e)^ PAVTI: pulmonary artery velocity time integral; ^f)^RVSP: right ventricular systolic pressure; ^g)^PCNA: proliferating cell nuclear antigen; ^h)^ CDK: cyclin-dependent kinase.

**Table S9.** Comparable data presentation for effect of exogenous irisin injection among hypoxia and SuHx mouse model.

| Parameters | Hypoxia mouse model | | | |  | SuHx mouse model | | | |
| --- | --- | --- | --- | --- | --- | --- | --- | --- | --- |
|  | Con | Irisin | Hypoxia | Irisin+hypoxia |  | Con | Irisin | SuHx | Irisin+SuHx |
| RVSP^a)^,mmHg | 18.71±3.80 | 15.61±3.41 | 36.76±5.35 | 26.62±2.20 |  | 19.63±3.12 | 15.60±2.99 | 39.43±3.87 | 23.44±3.18 |
| RV/LV+S^b)^ | 0.46±0.11 | 0.43±0.05 | 0.81±0.14 | 0.49±0.08 |  | 0.45±0.07 | 0.42±0.05 | 0.86±0.14 | 0.45±0.06 |
| PAVTI^c)^,mm | 37.06±1.89 | 39.68±1.90 | 34.38±1.24 | 39.64±1.82 |  | 37.23±1.73 | 40.79±2.14 | 30.16±3.71 | 38.11±2.24 |
| Pulmonary artery thickness/diameter% | 0.13±0.05 | 0.07±0.04 | 0.38±0.16 | 0.20±0.04 |  | 0.19±0.05 | 0.14±0.02 | 0.34±0.15 | 0.13±0.06 |

^a)^RVSP: right ventricular systolic pressure; ^b)^RV/LV+S: right ventricular to left ventricular + septum; ^c)^PAVTI: pulmonary artery velocity time integral.

**Table S10.** Primer sequence of related genes.

| Gene Name |  | Primer |
| --- | --- | --- |
| Homo-Irisin | Forward | GCATCAGAAACCAGCACACC |
|  | Reverse | CAGGTCTTGCCCTCACCTT |
| Homo-PDGF | Forward | CTCTTCCTGTCTCTCTGCTGCTA |
|  | Reverse | AGATCATCAAAGGAGCGGATCG |
| Homo-NEDD4 | Forward | AAACCGAATCCAGAAGCAAATG |
|  | Reverse | CTCCCAGTCCACACATAAGAAG |
| Homo-ENO1 | Forward | GCCGTGAACGAGAAGTCCTG |
|  | Reverse | ACGCCTGAAGAGACTCGGT |

**Table S11.** Interference RNA sequence of related genes.

| Gene Name |  | Interference RNA sequence |
| --- | --- | --- |
| Homo si-Irisin-1 | Forward | GGAGGAGGAUACGGAGUACTT |
|  | Reverse | GUACUCCGUAUCCUCCUCCTT |
| Homo si-Irisin-2 | Forward | CCCAAUAACAACAAGGAAATT |
|  | Reverse | UUUCCUUGUUGUUAUUGGGTT |
| Homo si-Irisin-3 | Forward | GCAAGCGUGUAAGAUGUUATT |
|  | Reverse | UAACAUCUUACACGCUUGCTT |
| Homo si-NEDD4 | Forward | GCACUAGUGCUAAAGGAUUTT |
|  | Reverse | AAUCCUUUAGCACUAGUGCTT |

**Table S12.** Antibodies in the article.

| Antibody | Manufacture |
| --- | --- |
| Irisin | Abclonal |
| PCNA | Cell signaling technology |
| βactin | Cell signaling technology |
| Goat Anti-Rat IgG H&L (HRP) | Abcam |
| Goat Anti-Mouse IgG H&L (Alexa Fluor® 488) | Abcam |
| Goat Anti-Rat IgG H&L (Alexa Fluor® 488) | Abcam |
| Goat Anti-Rabbit IgG H&L (Alexa Fluor® 594) | Abcam |
| Goat Anti-Rabbit IgG H&L (Alexa Fluor® 555) | Abcam |
| Goat Anti-Mouse IgG H&L (Alexa Fluor® 647) | Abcam |
| Goat Anti-American Hamster IgG H&L (Alexa Fluor® 647) | Abcam |
| EON1 | Abclonal |
| NEDD4 | Proteintech |
| Ki67 | Abclonal |
| CDK1 | Boster |
| CDK4 | Boster |
| CyclinD1 | PTMab |
| Ubiquitin | PTMab |
| α-SMA | Boster |
| Flag | Abclonal |

**Table S13.** Relevant parameters calculated in the process of eliminating outliers using the IQR method.

| Variable | Non-High risk | High risk |
| --- | --- | --- |
| Number | 55 | 38 |
| Q2 (Q1, Q3) | 3.145 (2.948, 3.472) | 2.768 (2.575, 3.156) |
| IQR^a)^ | 0.524 | 0.581 |
| Lower Limit | 2.162 | 1.7035 |
| Upper Limit | 4.258 | 4.0275 |

^a)^IQR: Interquartile Range.

Note: Q1: first quartile, Q2: second quartile; Q3: third quartile; IQR: interquartile range.

**Supplemental Figure legend**

**Figure S1.** Bioinformatics analysis of GEO datasets indicate reduced irisin expression in PAH patients. (A) The heatmap analysis of GSE144932 reveals the differential expression genes in PAH patients including down-regulated irisin/FNDC5. (B) The heatmap analysis of GSE113439 shows the differential expression genes including decreased irisin/FNDC5 in patients with PAH. (C) Volcano plot analysis of GSE144932. (D) Volcano plot analysis of GSE113439. (E) Decreased expression of irisin/FNDC5 in GSE144932. (F) Decreased expression of irisin/FNDC5 in GSE113439.

**Figure S2.** Irisin improves right heart function, pulmonary vascular fibrosis and metabolic changes in mice. (A and B) Western blot results show that irisin expression was reduced in lung tissue from hypoxia or hypoxia combined with SU5416-treated mice, whereas there is no change in expression in lung tissue treated with SU5416 alone(n=6). (C) Tricuspid annular plane systolic excursion (TAPSE) shows that irisin improves RV systolic function in hypoxia mice(n=6). (D to G) Western blot results show that irisin reduces collagen 1 expression in lung tissue from hypoxia or SuHx mice(n=6). (H to K) Masson's trichrome staining shows that irisin reduces perivascular collagen deposition in the lungs of hypoxia or SuHx mice(n=6). (L and M) Irisin significantly improves hypoxia-induced weight loss and reduces blood glucose in mice(n=6). In all graphs data are presented as mean ± SD. Data among 4 groups are compared by two-way ANOVA test followed by Tukey post hoc test for (A) to (M). ^**^*P*＜0.01, ****P*＜0.001, and *****P*＜0.0001 compared with AAV-null; ^&^*P*＜0.05, ^&&&^*P*＜0.001, and ^&&&&^*P*＜0.0001 compared with HYP+AAV-null. HYP, hypoxia; Su

Hx, hypoxia combined with SU5416.

**Figure S3.** Distribution and expression pattern of irisin in PDGF treated PASMCs. (A) Immunofluence images of irisin (Red) and SMA (Green) expression in PASMCs. (B) Immunofluorescence analysis indicates the well co-localization of irisin in PASMCs. (C) Increased mRNA of PDGF in pulmonary arteries of patients with PAH (n=6). (D and E) Decreased expression of irisin in PASMCs with PDGF treatment (n=3). (F) Enrichment analysis of GSE113439 illustrates the negative regulation of irisin on cell cycle. In all graphs data are presented as mean ± SD. Data between 2 groups are compared by independent-sample two-tailed Student’s t-test for (C). Data among 3 groups are compared by one-way ANOVA test followed by Tukey post hoc test for (D) and (E).

**Figure S4.** Irisin regulates proliferation, apoptosis and phenotypic switching in pulmonary artery smooth muscle cells. (A and B) EdU shows that irisin over-expression inhibites PDGF-induced PASMCs proliferation, whereas irisin knockdown has the opposite effect. (n=3). (C and D) Over-expression of irisin inhibites Bax expression and promotes Bcl-2 and Caspase-3 expression, whereas irisin knockdown has the opposite effect. (n=3). (E and F) Over-expression of irisin inhibites OPN expression and promotes α-SMA expression, whereas irisin knockdown has the opposite effect. (n=3). In all graphs data are presented as mean ± SD. Data among 6 groups are compared by two-way ANOVA test followed by Tukey post hoc test for (A) to (F).

**Figure S5.** Exogenous irisin treatment suppresses cell proliferation in human and mice PASMCs. (A and B) Western blot indicates reduced irisin expression in PDGF induced mice PASMCs (n=3). (C and D) Exogenous irisin decreases PCNA expression in a dose dependent manner (n=3). (E) CCK8 assay reveals exogenous irisin (100μg/ml) inhibites proliferation of PDGF induced human PASMCs (n=6). (F) CCK8 assay reveals exogenous irisin (100μg/ml) inhibites proliferation of PDGF induced mice PASMCs (n=6). (G and H) ELISA shows a decrease in intracellular irisin expression when treated with PDGF or when cell density increased. In all graphs data are presented as mean ± SD. Data between 2 groups are compared by independent-sample two-tailed Student’s t-test for (A) and (B). Data among 3 and above groups are compared by two-way ANOVA test followed by Tukey post hoc test for (C) to (H). 7500, 15000, 30000, 60000 represent different cell densities.

**Figure S6.** Screening for E3 ligases that bind to ENO1. **(**A) Schematic diagrams of molecular docking between irisin and ENO1. (B) Schematic diagrams of molecular docking among irisin, ENO1 and NEDD4. (C) Immunoprecipitation and Western blotting confirm no interaction between irisin and SYVN1. (D) Immunoprecipitation and Western blotting confirm no interaction between irisin and CBL.

**Figure S7.** Irisin regulates the proliferation of PASMCs via ENO1. (A) The EdU assay demonstrates that the enhancement of cell proliferation induced by irisin knockdown is reversed by ENO1 knockdown (n=3). (B) The EdU assay demonstrates that the reduction of cell proliferation induced by irisin over-expression is reversed by ENO1 over-expression (n=3). In all graphs data are presented as mean ± SD. Data among 4 groups are compared by two-way ANOVA test followed by Tukey post hoc test for (A) to (B).

**Figure S8.** Irisin regulates proliferation and cell cycle of PASMCs by targeting ENO1. (A and B) The Ki67 assay shows that knockdown of ENO1 attenuates the proliferative effect of irisin on PASMCs, and subsequent re-expression of ENO1 restores this effect. (n=3). (C and D) The EdU assay shows that knockdown of ENO1 attenuates the proliferative effect of irisin on PASMCs, and subsequent re-expression of ENO1 restores this effect. (n=3). (E and F) Knockdown of ENO1 attenuates the increase in PCNA, CDK1, CDK4, and CyclinD1 protein expression induced by irisin knockdown, and these effects are reversed by subsequent re-overexpression of ENO1. (n=3). (G) The CCK8 assay shows that knockdown of ENO1 attenuates the proliferative effect of irisin on PASMCs, and subsequent re-expression of ENO1 restores this effect. (n=6). In all graphs data are presented as mean ± SD. Data among 4 groups are compared by two-way ANOVA test followed by Tukey post hoc test for (A) to (G).

**Figure S9.** Effect of different concentrations of irisin on hypoxia-induced pulmonary hypertension. (A) Quantitative analysis reveals a significant reduction in RVSP in mice administered 250 µg/kg of irisin (n = 6). (B) Decreased RV/LV+S ratio in mice administered 250 µg/kg of irisin (n = 6). (C) Increased PAVTI in mice administered 250 µg/kg of irisin (n = 6). (D and E) Western Blot demonstrates the decreased protein expression of PCNA, CyclinD1 and CDK1, CDK4 in mice administered 250 µg/kg of irisin (n = 6). In all graphs data are presented as mean ± SD. Data among 4 groups were compared by two-way ANOVA test followed by Tukey post hoc test for (A) to (E).

**Figure S10.** Validation of over-expression or knock down efficiency of related genes. (A) mRNA expression in PASMCs with irisin over-expression (n=6). (B and C) Protein expression efficiency in PASMCs with irisin over-expression (n=3). (D) mRNA expression in PASMCs with irisin knocked down (n=6). (E and F) Protein expression efficiency in PASMCs with irisin knocked down (n=3). (G) mRNA expression in PASMCs with ENO1 over-expression (n=6). (H and I) Protein expression efficiency in PASMCs with ENO1 over-expression (n=3). (J) mRNA expression in PASMCs with NEDD4 knocked down (n=6). (K and L) Protein expression efficiency in PAMSCs with NEDD4 knocked down (n=3). (M) mRNA expression in PASMCs with ENO1 knocked down (n=6). (N and O) Protein expression efficiency in PASMCs with ENO1 knocked down (n=3).In all graphs data are presented as mean ± SD. Data between 2 groups are compared by independent-sample two-tailed Student’s t-test for (A) to (C), and (G) to (L). Data among 4 and above groups are compared by one-way ANOVA test followed by Tukey post hoc test for (D) to (F).

**Figure S1**


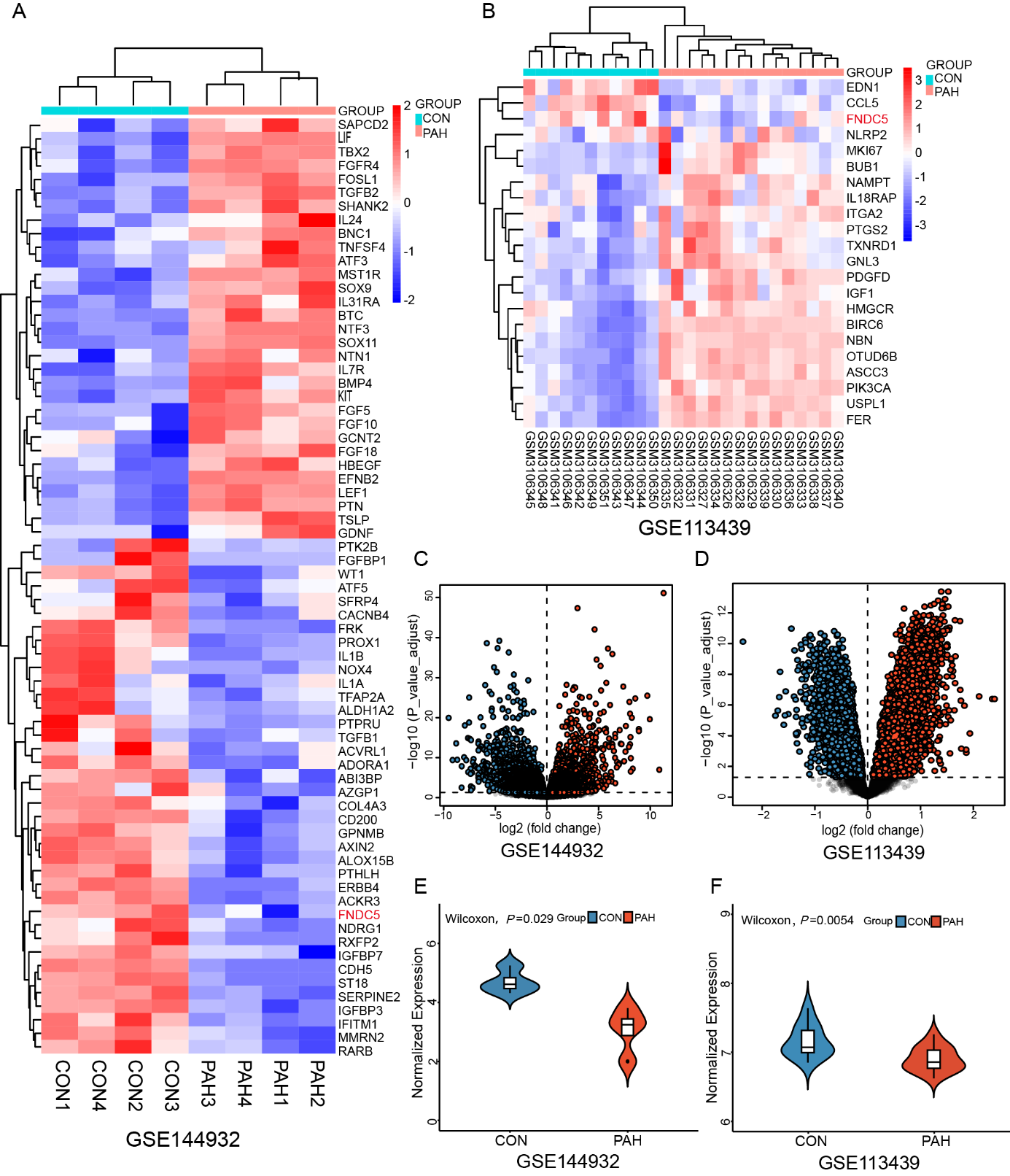


**Figure S2**


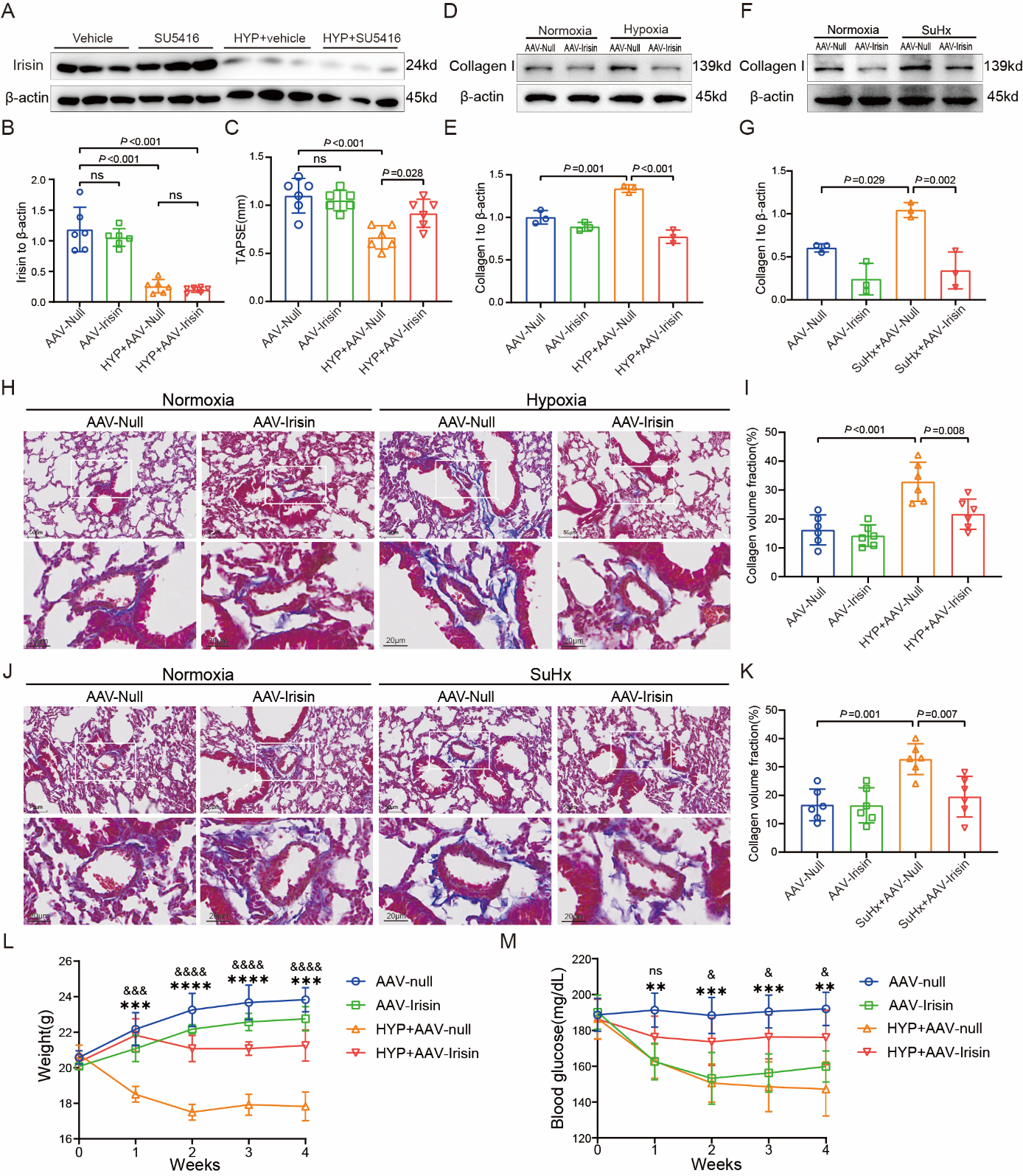


**Figure S3**


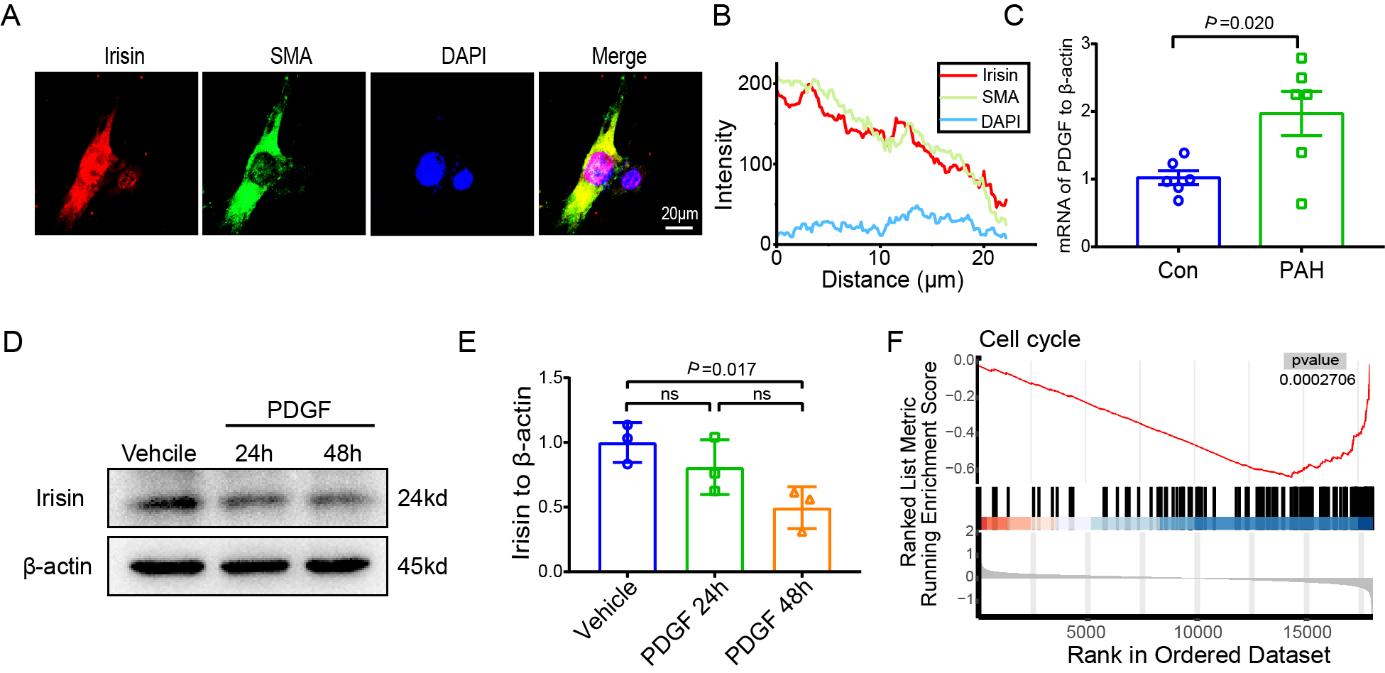


**Figure S4**


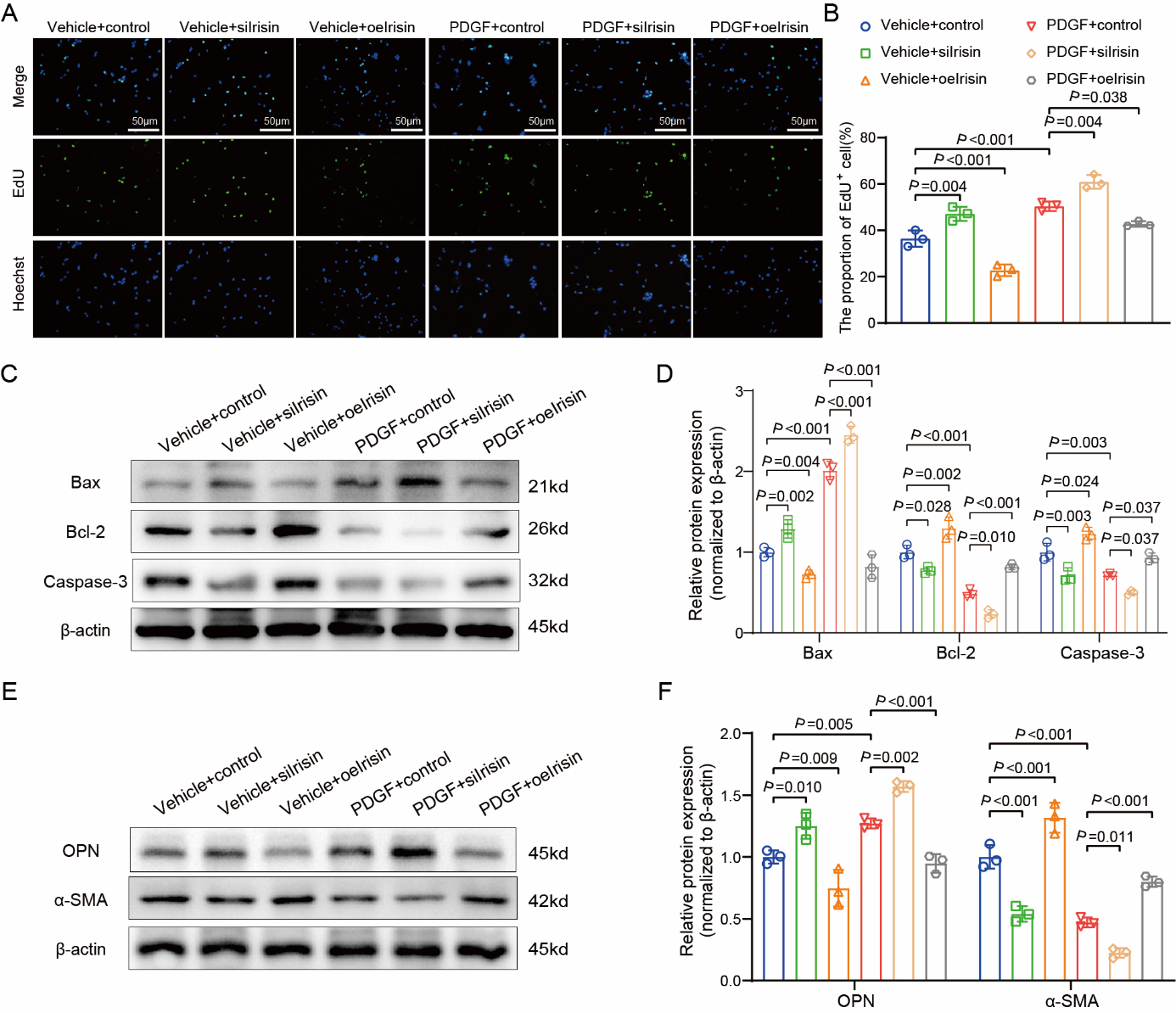


**Figure S5**


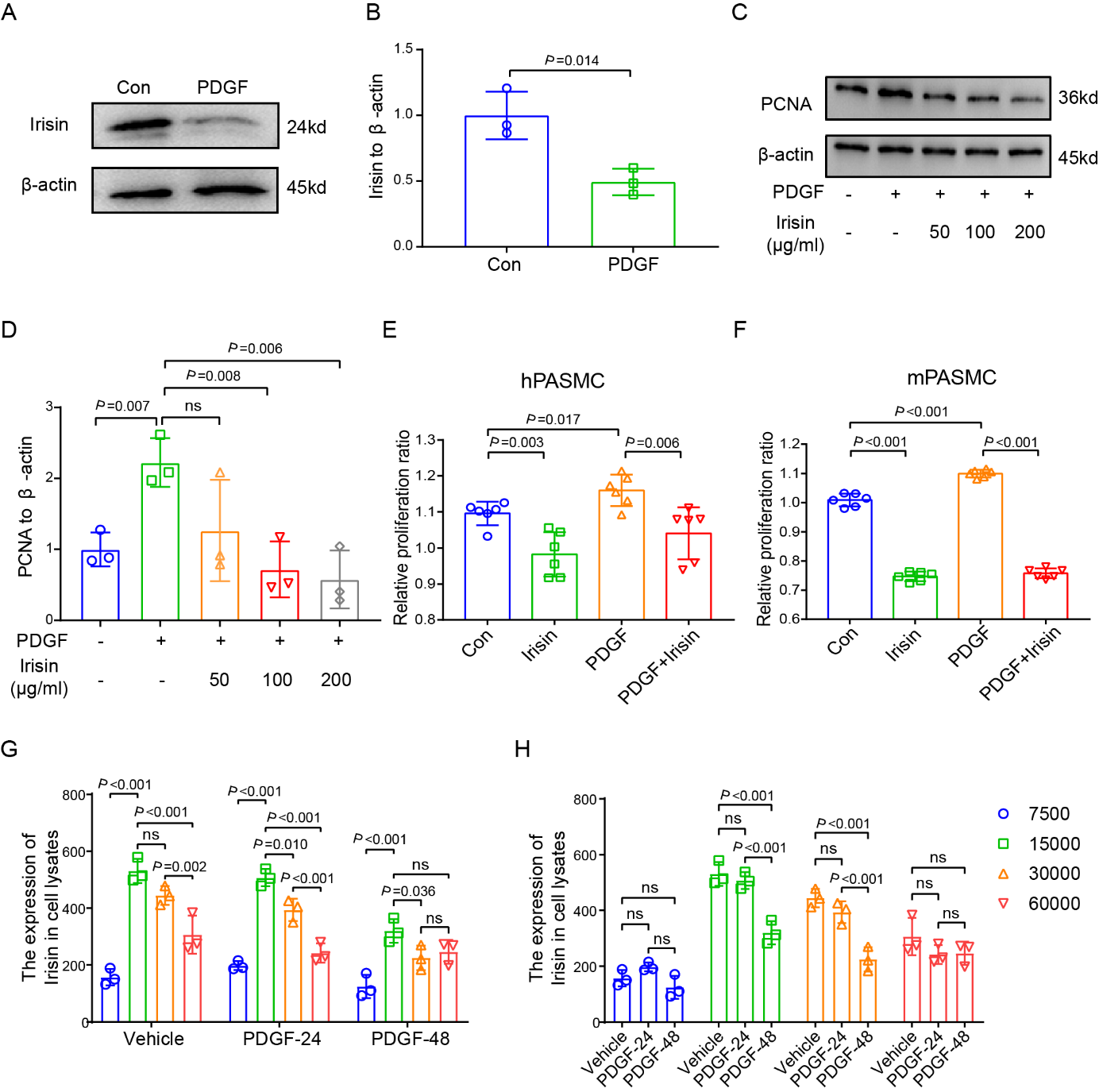


**Figure S6**


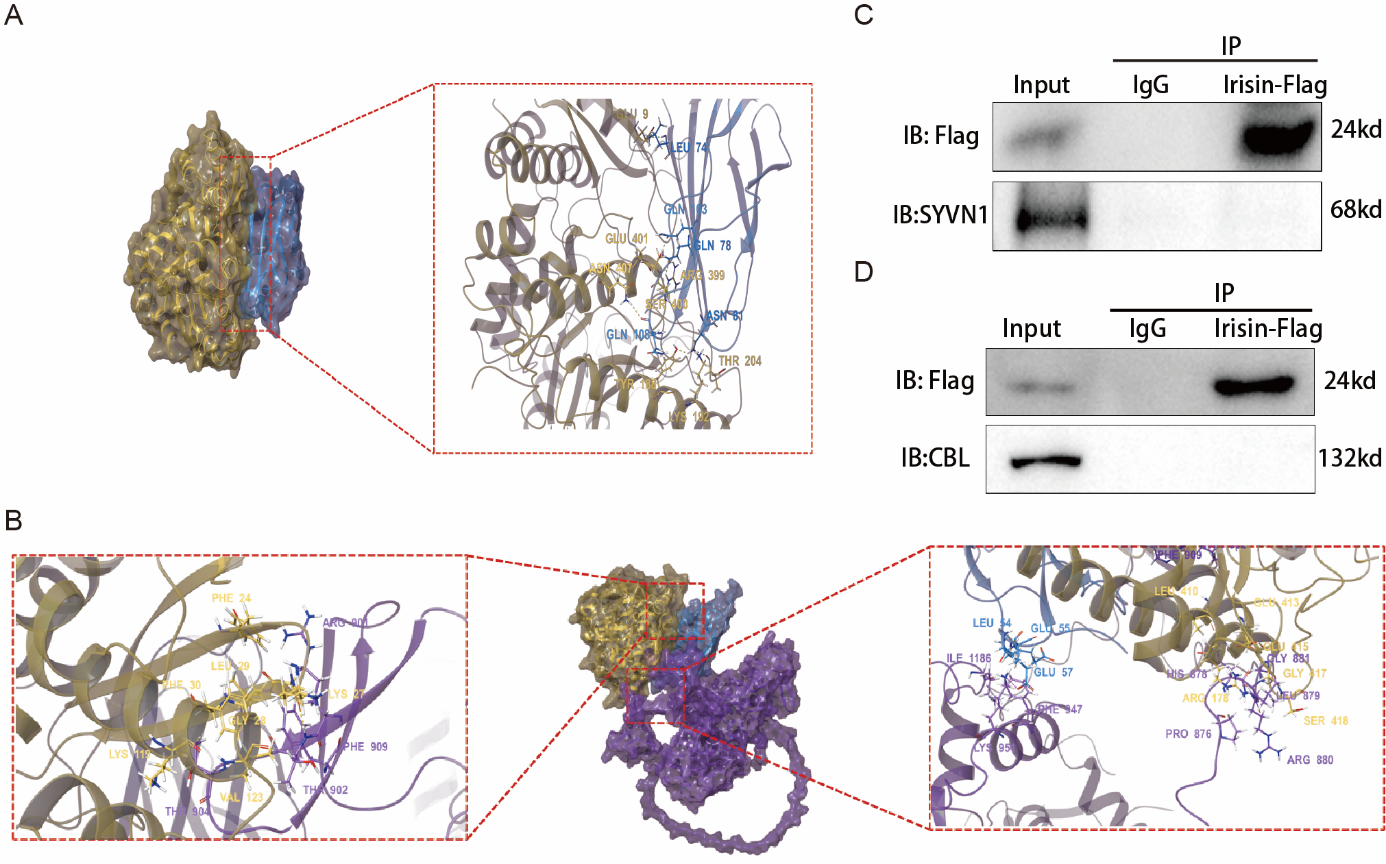


**Figure S7**


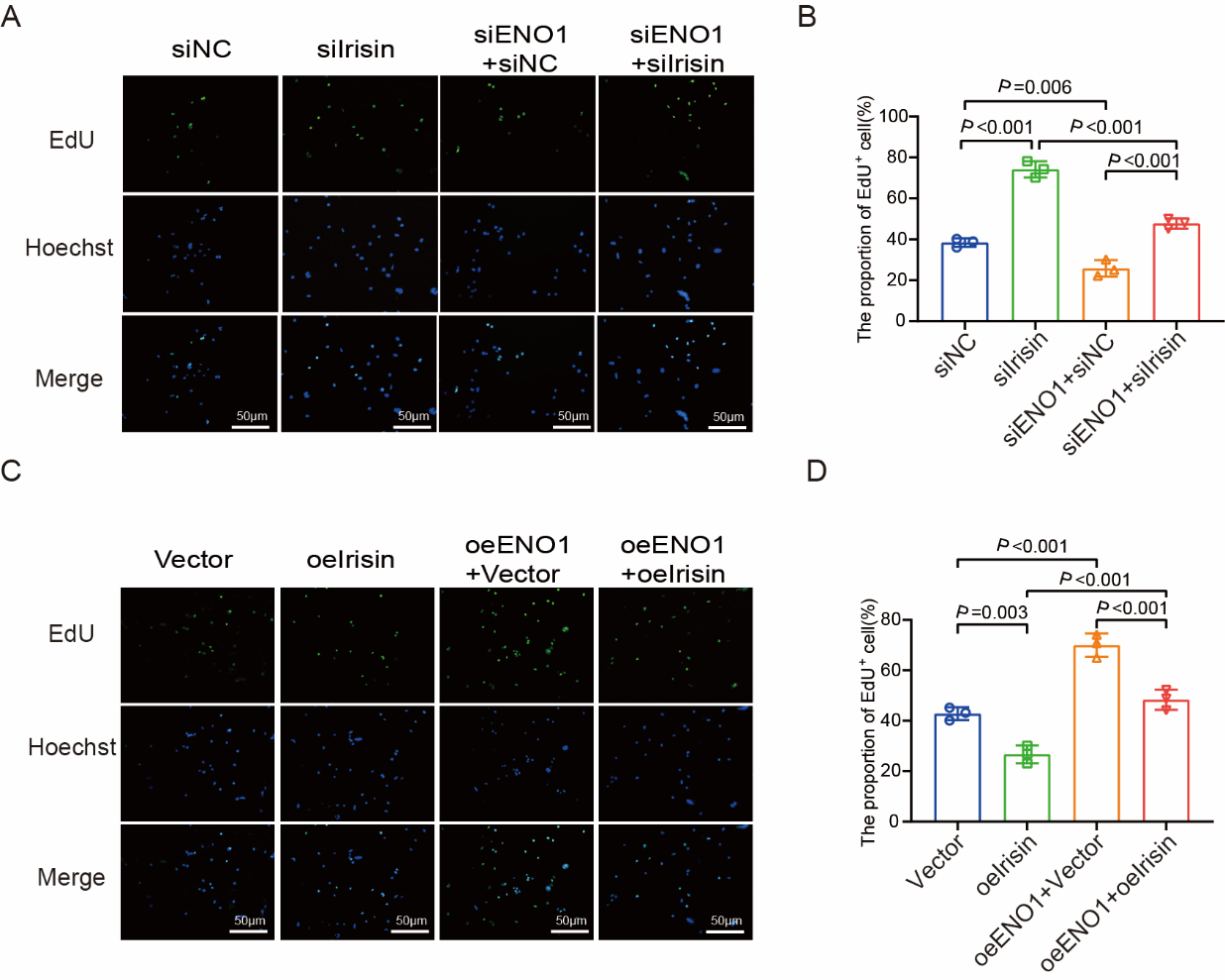


**Figure S8**


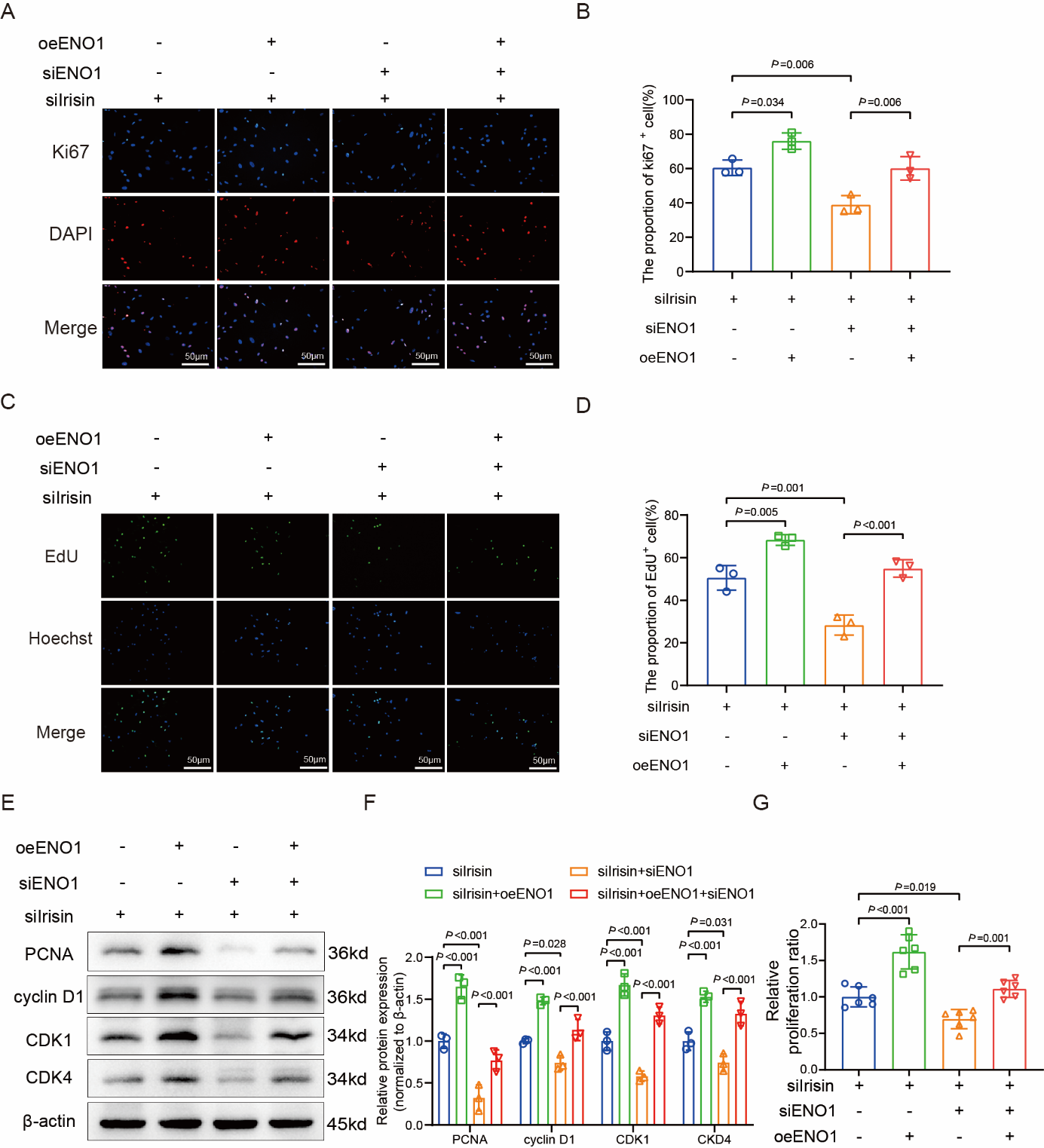


**Figure S9**


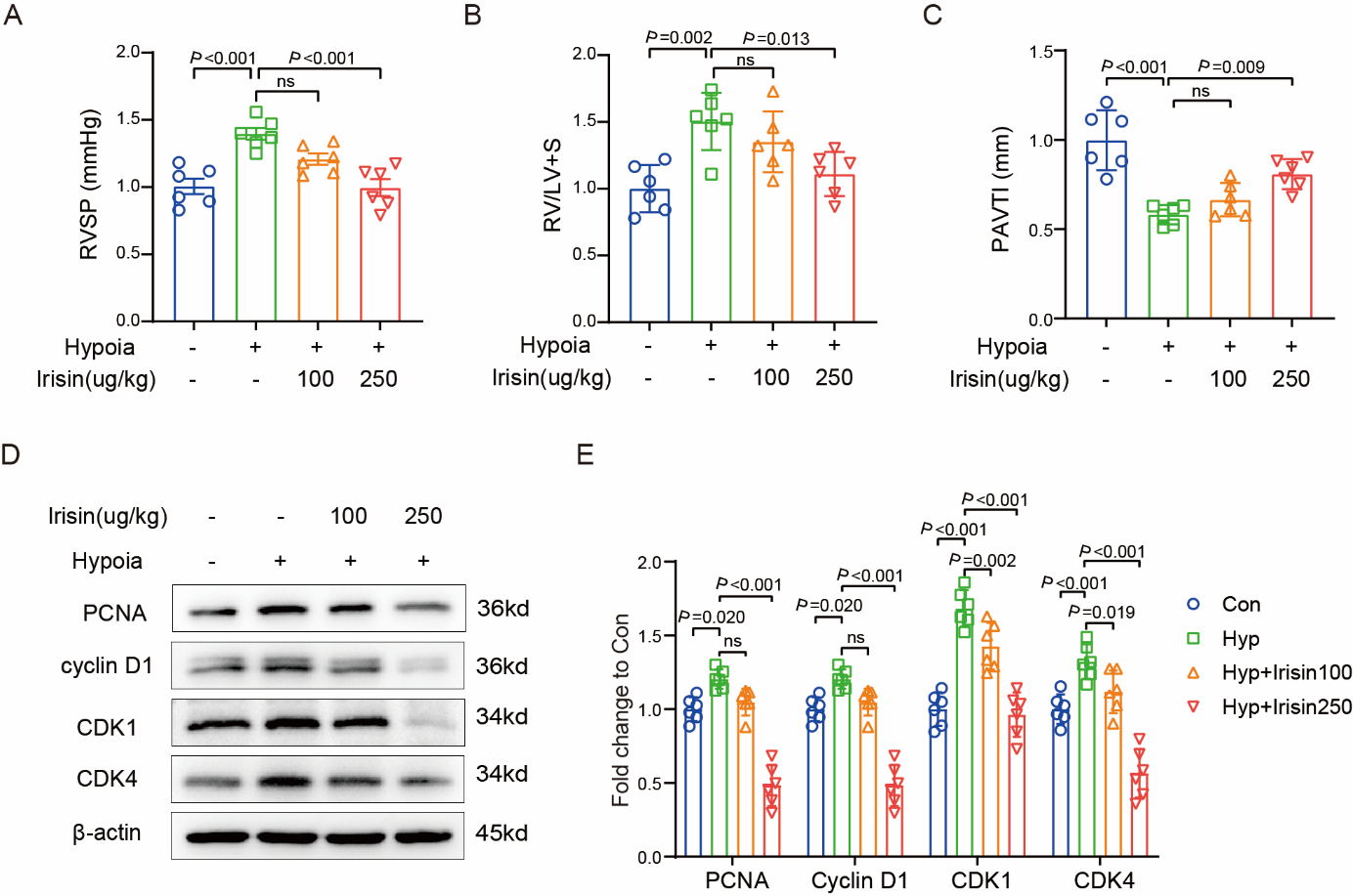


**Figure S10**


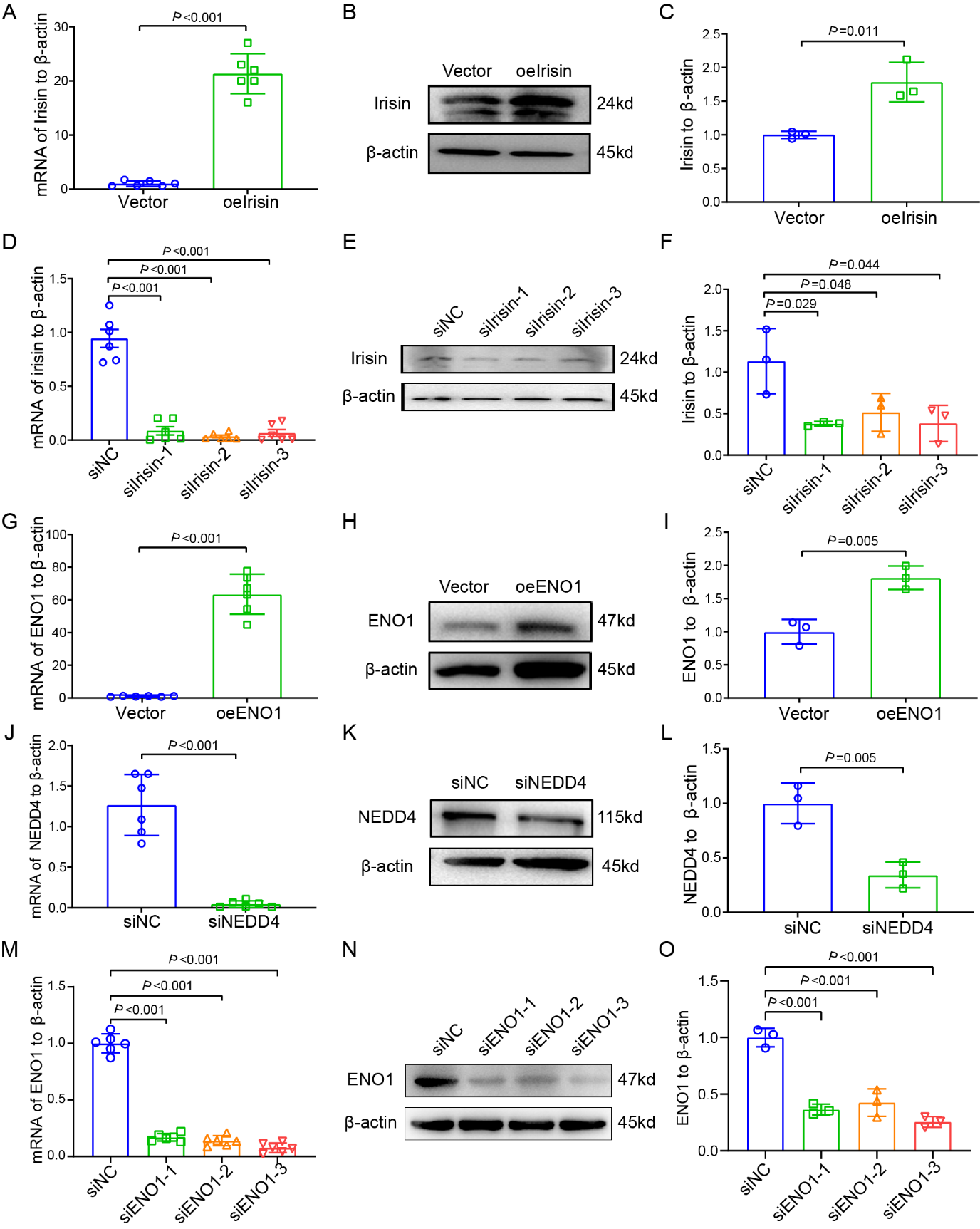

Supplement: Supplementary file 1 — Supporting Information [file ADVS-12-e00096-s001.docx]
